# Genomic epidemiology and phylogeographic reconstruction of West Nile virus 2 in Italy from 2011 to 2023

**DOI:** 10.1016/j.onehlt.2025.101310

**Published:** 2025-12-24

**Authors:** Carla della Ventura, Maya Carrera, Francesco Defilippo, Davide Lelli, Chiara Nogarol, Maria Lucia Mandola, Alessia Lai, Annalisa Bergna, Francesca Moroni, Ana Moreno, Gianguglielmo Zehender

**Affiliations:** a Department of Biomedical and Clinical Sciences, University of Milan, 20157 Milan, Italy; b CRC-Coordinated Research Center "EpiSoMI", University of Milan, 20157 Milan, Italy; c Istituto Zooprofilattico Sperimentale della Lombardia e dell'Emilia-Romagna (IZSLER), 25124 Brescia, Italy; d Istituto Zooprofilattico Sperimentale del Piemonte, Liguria e Valle d'Aosta (IZSPLV), 10154 Torino, Italy; e Laboratory of Medical Microbiology and Virology, University of Insubria, 21100 Varese, Italy

**Keywords:** West nile virus, Lineage 2, Whole genome sequencing, Epidemiology, Phylogeographical approach

## Abstract

Since its introduction to Europe in 2004, West Nile Virus Lineage 2 (WNV-2) has become endemic, with Italy reporting the highest number of cases each season. In 2022, WNV infections in Italy exceeded those recorded during the major 2018 outbreak—the largest ever reported in Europe. This study investigates the genomic epidemiology of WNV during the 2022 and 2023 transmission seasons.

We analyzed 123 environmental samples from wild birds and mosquito pools collected between May and October 2022–2023 in northwestern Italy. All but one sample belonged to Lineage 2; lineage 1 was detected in two bird samples, with one showing co-infection. A total of 98 complete genomes were sequenced.

Phylogeographic reconstruction indicated the origin of the main European clade in Hungary in 2004, with introduction into Italy between 2009 and 2010. Most Italian genomes clustered within a single highly supported subclade, with one sampled in the Marche region in 2011 as the outgroup. Continuous phylogeographic analysis suggested the Italian WNV-2 clade originated in 2009 in the area between Emilia-Romagna and Lombardy, followed by east-west spread during 2022–2023.

Several mutations were identified, including F49L in the NS2A gene linked to neuronal tropism, and M184V in the NS4B gene, associated with increased pathogenicity.

Our results highlight how integrated genomic surveillance of WNV, combining whole genome sequencing and phylogenetic analyses to environmental samples, can support One Health approaches for early detection and risk assessment of arboviral transmission.

## Highlights

• Phylogeography indicates a single WNV-2 entry into Italy in 2009, followed by eastward and westward spread across Po Valley.
• Regional clusters and positively selected mutations show co-circulating WNV populations with varying pathogenic potential.
• Integration of genomic, temporal, and spatial data shows the importance of real-time molecular surveillance for WNV control.

## 1 Introduction

West Nile Virus (WNV) is a positive-sense single-stranded RNA virus, belonging to the Japanese encephalitis virus (JEV) serocomplex of the *Flaviviridae* family, genus Orthoflavivirus (renamed in 2023) [1], including more than 50 species of viruses transmitted by vectors [2,3].

WNV infection is associated with mild febrile syndrome in about 20 % of infected humans, known as West Nile fever (WNF), while 80 % of the infections are asymptomatic. In less than 1 % of cases, the disease occurs in the neuroinvasive form (West Nile Neuroinvasive Disease-WNND), with an overall case fatality rate being around 10 % (among patients with WNND) but can be higher in elderly individuals [4].

The virus is maintained in nature through an enzootic cycle, involving several wild bird species belonging to different orders, most frequently represented by Passeriformes [5], and mosquitoes of the *Culex* genus. Humans, horses, and other vertebrates (reptiles, amphibians, mammals) are in general dead-end hosts [6]. WNV represents the most widespread cause of vector-borne viral encephalitis, being present in all continents but Antarctica. Because its transmission occurs at the interface between wildlife, vectors and humans, WNV is a paradigmatic One Health issue, where environmental and animal surveillance can provide early warning signals of human risk.

Up to now, among the nine evolutionary lineages identified, only two (WNV-1 and WNV-2) are known to be pathogenic in humans. WNV-1 (in particular the sublineage 1a) is widespread in all continents representing the main lineage circulating in Europe until 2004, when the lineage WNV-2, previously confined to sub Saharan Africa, was identified for the first time from a bird of prey in Hungary in 2004 [7], and spread to the eastern Austria and several European countries [8,9], causing large outbreaks in Northern Greece in 2010 [10]. Nowadays, WNV-2 is the most prevalent WNV lineage circulating in the continent. In 2018 WNV-2 caused the largest outbreak in Europe with 1503 human cases, 69 % of which were neuroinvasive diseases, and 181 deaths reported in 11 European countries [4,11] [a].

In Italy, West Nile of sublineage 1a (WNV-1a) was reported for the first time in 1998 in Tuscany, causing cases of neurologic disease and deaths among horses The first human cases were documented in August 2008 in three Italian regions: Emilia Romagna, Veneto and Lombardy [12]. The first evidence of WNV-2 in Italy was in 2011 with two sporadic cases, in Ancona province and in Sardinia, co-circulating with WNV-1a [13,14]. In the following years, WNV-2 rapidly spread all over the country and replaced WNV-1, [15,16]. From June to November 2018 a first largest epidemic occurred in Northern Italy, causing 230 neuroinvasive diseases and 42 deaths (CFR: 18.2 %) [a] [11]. After a few years in which only WNV-2 was detected, WNV lineage 1 re-emerged in Italy around 2020–2021, initially in southern regions and subsequently in north-eastern Italy, where it co-circulated with WNV-2 and contributed to the large outbreaks reported in 2022 [15,16].

To date, Italy is the European country with the largest number of WNV cases [b].

The aims of this study were to develop a protocol for WNV-2 amplification and Whole Genome Sequencing (WGS), and to evaluate the phylogeny of the virus circulating in Northern Italy during 2022 and 2023 seasons (from late spring to early fall) in environmental samples, including birds and mosquitoes.

## 2 Materials and methods

### 2.1 Birds and mosquito collection

Mosquito collection was performed in georeferenced stations within of the national surveillance system targeting West Nile virus. All these stations were monitored by modified CDC traps baited by CO2 (CO2 traps) [17]. Mosquitoes were identified to species level using morphological characteristics according to Becker N, et al. [18]. Mosquitoes (both male and female specimens) were pooled according to date, location and species, with a maximum number of 100 individuals per pool, without separating individuals by sex.

Ornithological collection was performed through active surveillance (capture and killing of resident “reservoir” birds of selected species) and through passive surveillance (identifying episodes of abnormal wildlife mortality or analyzing animal death in wildlife refuge centers). Carcasses of magpie (*Pica pica*), hooded crow (*Corvus corone cornix*), and Eurasian jays (*Garrulus glandarius*) were collected and delivered to the laboratory by rangers and hunters. These species are considered pests for crops and, therefore, are under population control programs, authorized yearly by the National Institute for Wildlife (ISPRA). The sampling from passive surveillance mainly involved birds belonging to the orders Accipitriformes, Charadiiformes, Columbiformes, Falconiformes, and Passeriformes.

Starting from the sampling conducted for the integrated surveillance of West Nile virus in the seasons 2022 and 2023, a subset of samples that tested positive for WNV lineage 2 was collected. In particular, the Sperimental Zooprophylactic Institute of Lombardy and Emilia-Romagna (IZSLER) selected 108 samples (76 homogenates of bird organs and 32 mosquito pools) and the Sperimental Zooprophylactic Istitute of Piedmont, Liguria and Valle d'Aosta (IZSPLV) selected 15 samples (all homogenates of bird organs).

### 2.2 Sample processing

For mosquitoes, analyses for WNV genome detection were performed only on *Culex* spp. as these species are considered a highly competent vector for WNV [19]. Each pool of *Culex* spp. was homogenized in phosphate-buffered saline (PBS) in a 2-mL microtube with coppered microspheres and then stored at −80 °C until viral screening. For birds, portions of brain, spleen, heart, and kidney were collected from each animal during necropsy. The tissue pool from each bird was homogenized with Stomacher® in PBS at a 1:10 dilution.

RNA from IZSLER was manually extracted from bird homogenates and mosquito pools by using IndiMag Pathogen kit (Indical Bioscience Gmbh. Leipzig, DE) according to the manufacturer's instructions, while the RNA from IZSPLV was extracted automatically using the Maxwell® RSC simplyRNA tissue procedure (Promega Corporation, Madison, WI, USA) (see Supplementary Material). The samples from IZSPLV were analyzed with a TaqMan One-Step RT-PCR (Applied Biosystems, Foster City, CA) for WNV according to Tang et al. [20]. A multiplex quantitative real-time reverse transcription polymerase chain reactions (qRT-PCR) for simultaneous detection of WNV-1, WNV-2 as well as a real time RT-PCR for Usutu virus [21,22] were performed on samples from IZSLER for typing.

### 2.3 Sample processing for whole genome sequencing

RNA from all the samples was manually extracted from bird homogenates (see Supplementary Material) and mosquito pools by using QIAMP viral RNA mini-Kit (Qiagen GmbH, Germany), according to the manufacturer's instructions. After the extraction, RNA was quantified on Infinite M200 Pro instrument with NanoQuant plate (Tecan, Trading AG, Switzerland) and only the RNA of positive WNV-2 samples has been reverse-transcribed to cDNA using the LunaScript® RT SuperMix Kit (New England Biolabs, Ipswich, MA) and amplified by two home-made protocol using primer pools generating 400 bp amplicons or specific primers, when the Ct (cycle threshold) value of the qPCR previously performed was ≤33. The primers for each pool were generated on a dataset of whole genome sequences of WNV-2 from the Genbank database [c] (acc. n° KF647252.1, MW142226.1, MW142227.1, OK239663.1, MT863560.1, MT863561.1, MW561633.1, OK239665.1, OK239671.1, OK239672.1, OK239669.1, MH021189.1, MF984346.1, MF984347.1, MF984348.1, MF984350.1, MF984351.1, MF984352.1, KU206781.1, MF984337.1, MF984338.1, MF984339.1, MF984340.1, MF984342.1, MF984343.1, MH244512.1, MH244513.1, KF647250.1, KT757322.1, KT757323.1, KF179639.1, KY594040.1, HQ537483.1, KF647250.1) with the PrimalScheme online tool, selecting an amplicon size of 400 bp in two pools, a minimum base frequency of 0.01, and a target overlap of 40 bp as default [d] (Supplementary Table S1). The PCR reaction was performed in a 25 μL of mix for each pool containing 6 μL of template cDNA, 2.5 μL of each 10 μM primer pool, 12.5 of Q5® Hot Start High-Fidelity 2× Master Mix (New England Biolabs, Ipswich, MA) and 4 μL of Nuclease-free water. Thermocycling conditions consisted of 30 s at 98 °C, followed by 35 cycles of 95 °C for 15 s and 65° for 5 min. The results of the PCR were checked on the 4200 TapeStation System (Agilent, Santa Clara, US), to detect the exact amplified peak at 400 bp and then purified using a 1.8× volume of Agencourt AMPure XP Beads (Beckman Coulter, Brea, CA). Amplicons were pooled, diluted to equal concentration and libraries were prepared using Illumina DNA Prep and IDT ILMN DNA/RNA Index kit (Illumina, San Diego, CA). Library concentration was determined with the Invitrogen Quant-iT Picogreen dsDNA assay (Fisher Thermo Scientific, Waltham, MA). Resulting libraries were normalized and pooled for sequencing using a 2 × 200 cycle paired-end sequencing protocol. Consensus sequences were generated mapping FASTQ files to a reference sequence (NC_001563.2) with Geneious Prime software v. 11.1 [e].

### 2.4 Dataset preparation

A dataset of 334 whole genome sequences was built including European WNV-2 complete genome (WNV-2 European dataset) sequences available on public databases (Genbank) with collection date from 2004 to 2023. We included all WNV-2 sequences available on Genbank and Pathogen-Portal (Pathoplexus) [f] at the time of analysis. Only whole genome sequences and with clearly annotated lineage, collection date, and geographic location were retained. Countries of collection were Greece (*n* = 60), Austria (*n* = 20), Hungary (*n* = 5), Czech Republic (*n* = 4), Slovakia (n = 4), Serbia (n = 4), Germany (n = 4), Bulgaria (n = 2), Spain (n = 2), Russia (n = 2), Kosovo (*n* = 1) and Italy (*n* = 226). Therefore, Italian subset included 226 sequences, of which 96 newly characterized and 61 previously characterized [11].

In addition, the Italian subset was cut for the E gene portion (*n* = 220), including only strains with geographical coordinates. This dataset comprises both newly generated whole genomes and previously sequenced partial E gene sequences. GenBank accession numbers, collection locations, and countries for all reference sequences are reported in Supplementary Table S2.

Sequences were aligned and edited using the ClustalW algorithm implemented in BioEdit v. 7.2.6.1 program [g] or by manual editing when necessary. All genomes were trimmed to the same length of 10,053, corresponding to the size of the viral polyprotein. To detect the presence of recombinant sequences, the dataset was screened using RDP5 (Recombination Detection Program) [23], that includes several methods for investigating primary recombination events, i.e. RDP [24], BOOTSCANning [25], GENCOV [26], MAXCHI, CHIMAERA [27], SiSCAN [28] and 3 SEq. [29]. Only sequences detected by at least 5 methods out 7 were considered recombinants. Additional confirmation of recombination presence was evaluated using GARD (Genetic Algorithm for Recombination Detection) method on the Datamonkey server [30] [h].

### 2.5 Phylogenetic and discrete phylogeographic analysis

Phylogenetic model TIM2 + F + I (AC = AT, CG = GT and unequal base frequencies + empirical base Frequencies + Invariant sites) was estimated and selected using the ModelFinder tool [31], implemented in IQ-TREE v.1.6.12 [i] [32].

The phylogeny of the European dataset was reconstructed with the Maximum-Likelihood method, using IQ-TREE v.1.6.12. The reliability of clades was established based on bootstrap values of internal nodes ≥60 % (after 1000 replications). The generated tree was visualised using Tempest v1.5 program to find out the year of the common ancestor (tMRCA, the Most Recent Common Ancestor) and the correlation coefficient (R-squared, R^2^).

The Nextstrain platform, the bioinformatics pipeline for the phylodynamic analysis and interactive visualization, was used to obtain a dated tree. File preparation, alignment, and phylogenetic analysis were performed using the standard Nextstrain build workflow with the augur and auspice toolchain, using the publicly Zika virus tutorial available on the Nextstrain website [j], which provides all commands and parameters used.

The phylogenetic tree generated on the Italian complete genome subset was used for the identification of significative Italian clusters using Cluster Picker v.1.2.3124 software, which allows clusters of potentially epidemiologically related sequences to be found within a phylogenetic tree [33].

Only clusters with a bootstrap values of internal nodes greater than 60 % and an average genetic distance between sequences less than 1 % as thresholds were considered.

### 2.6 Continuous phylogeography

On the Italian complete genome and E gene subsets, the REL molecular clock and a Bayesian skyline plot (BSP) demographic model were used. The substitution model was selected in J Modeltest [27] using the Akaike (AIC) and Bayesian information criteria (BIC) and a “decision-theoretical performance-based” approach [32]. The model selected for subsets was GTR + I + G (General Time Reversible) with a proportion of invariant sites and gamma distribution.

The generalised path sampling and stepping stone sampling marginal likelihood estimators [34] were used to determine the best fitting clock and demographic models. Four simple parametric models (constant, exponential, expansion and logistic population growth) and the Bayesian skyline plot (BSP), skyride and skygrid were compared as coalescent models under both a strict and a relaxed (uncorrelated log-normal, UCLN) clock [35].The final tree with the highest posterior probability product (maximum clade credibility) was selected based on the maximum posterior probability (pp) value after at least 10 % burn-in using the Tree Annotator v.10.4 software (included in the BEAST v 1.10.4 package).

For the continuous phylogeography analysis, the strict Brownian diffusion model and several Relaxed Random Walk models were compared by Bayes factor estimation using Path Sampling and Stepping-Stone sampling, and the RRW model with Gamma distribution was selected as the best tree prior (see Table 1).

**Table 1:**
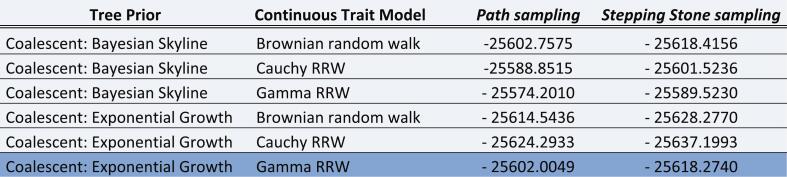
Comparison of models with respective path sampling and stepping stone sampling values. In dark, highlighted the winning model for the continuous phylogeographic analysis.

The uncertainty of the estimates obtained was indicated with 95 % Highest Posterior Density (HPD) intervals. Both trees were displayed and edited using FigTree v. 1.4.4 [k]. The virus spread trends were represented and visualised with the program SpreaD3 v1.0.7 [36].

For the investigation of the dispersal history and dynamics of viral lineages, the R package, Seraphim, was used. The package was also used to extract and summarise spatio-temporal information from the posterior distribution of phylogeographic trees, and to estimate dispersal statistics (e.g. branch-specific distances, diffusion coefficients and dispersal velocities) and to map continuous phylogeographic trees [l] [37]. Given the scarcity of whole genomes whose spatial coordinates of the sampling location are known, especially in the past years when single gene sequencing was more common, we decided to show only the results of continuous phylogeography analysis of the E gene dataset, which included a larger number of sequences.

Details on all sequences included in each dataset, together with their accession numbers, collection dates, locations and the analyses in which they were used, are provided in Supplementary Table S2.

### 2.7 Selective pressure

The selective pressure analysis on newly characterized samples (*n* = 98), was performed considering genes coding for structural (pre-M/M and E) and non-structural (NS1, NS2A, NS2B, NS3, NS4A, NS4B and NS5) proteins, to identify codon sites potentially subject to positive or purifying selection. The analysis was conducted on the Datamonkey server. The used approaches were: Single Likelihood Ancestor Counting (SLAC), Fixed-Effects Likelihood and internal Fixed-Effects Likelihood (FEL/iFEL), Fast, Unconstrained Bayesian AppRoximation (FUBAR) and adaptive Branch-Site Random Effects Likelihood (aBSREL) [38]. Unless otherwise specified, models were run under a general time-reversible (GTR) nucleotide substitution model with a proportion of invariant sites and gamma-distributed rate variation, using the Datamonkey default settings. Codon sites were considered to be under positive selection when supported by FEL/iFEL with *p* < 0.1 and/or by FUBAR with posterior probability >0.9, and when results were consistent across at least two methods.

## 3 Results

### 3.1 WNV evolutionary lineage assignment

The RT-PCR analysis of the environmental samples (birds and mosquitoes' pools) revealed the presence of lineage 2 in the 122/123 (99.2 %) of the samples analyzed, while lineage 1 was observed in two birds (1.6 %): one of which resulting coinfected by both lineages. Moreover, 12 samples (1 mosquitoes' pool collected in 2022 and 11 birds collected in 2023), also showed the presence of Usutu RNA virus. The results are summarized in Table 2.

**Table 2:** Viral classification of samples by RT-Real Time PCR.

Classification	Bird (*n* = 91)		Mosquito (*n* = 32)		Total (*n* = 123)
	2022 (*n* = 41)	2023 (*n* = 50)	2022 (*n* = 21)	2023 (*n* = 11)	
WNV L1	2⁎ (4.9 %)	–	–	–	2 (1.6 %)
WNV L2	40* (97.6 %)	50⁎⁎ (100 %)	21⁎⁎⁎ (100 %)	11 (100 %)	121 (98.3 %)
USUV	–	11⁎⁎ (22 %)	1⁎⁎⁎ (4.8 %)	–	12 (9.8 %)

⁎ One bird was co-infected with WNV-1 and WNV-2.

⁎⁎ Eleven birds were co-infected with WNV-2 and USUV.

⁎⁎⁎ One mosquito pool tested positive for both WNV-2 and USUV.

### 3.2 Quality of WNV-2 complete genome sequences amplified by primer pool protocol

As shown in Supplementary Table S3, a total of 98/123 (79.7 %) WNV-2 complete genome sequences were obtained. The mean number of reads obtained was 419,500 (min 7950 - max 3,280,530). The mapping of the reads with the reference genome resulted in an average sequence coverage of 7455,02 (min 0 - max 169,678). The mean confidence, provided by the base calling program, had a value of 35,8, with a quality score (Q-SCORE) percentage of 96,1 % (Q20) and 92,9 % (Q30).

### 3.3 Phylogenetic analysis of the European complete genome WNV-2 dataset

Recombination analysis did not detect the presence of any significant recombination event.

**Fig. 1:**
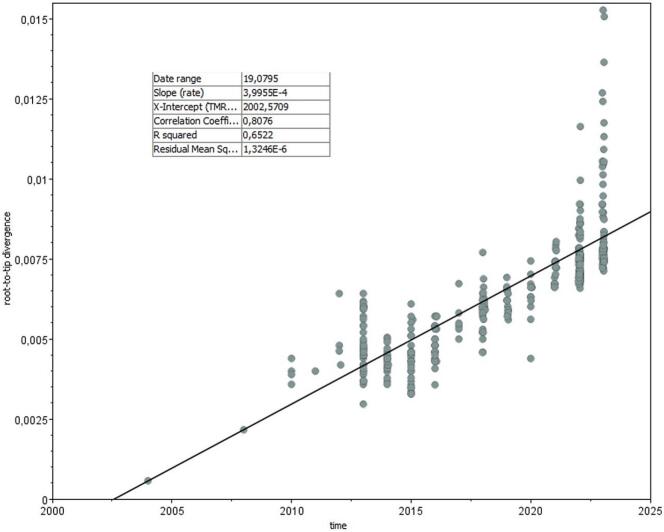
Root-to-tip regression analysis of the entire WNV-2 European dataset.

Root-to-tip regression analysis of the entire WNV-2 European dataset showed significant association between genetic distances and sampling times, with a correlation coefficient of 0.8076 and a coefficient of determination (R2) of 0.6522 (Fig. 1).

**Fig. 2:**
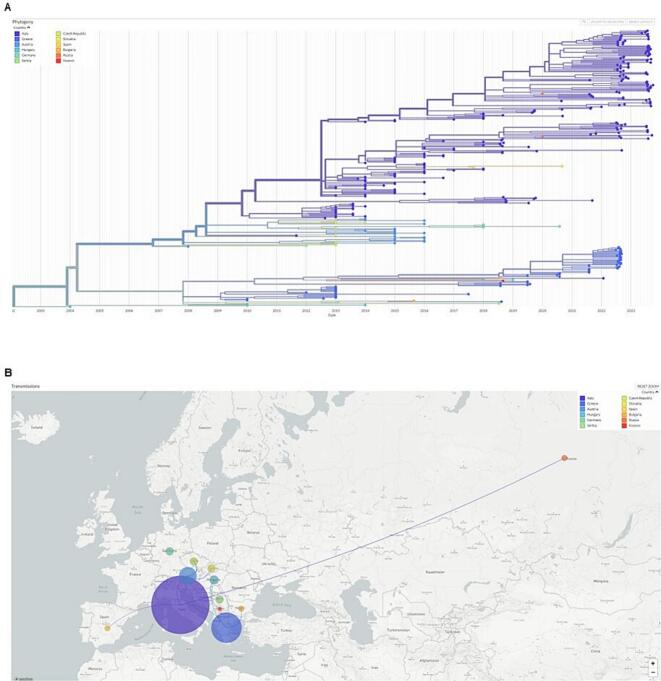
Maximum-Likelihood tree (A) and the geographic resolution of WNV-2 circulation (B) generated on the Nextstrain platform.

Fig. 2 shows the Maximum-Likelihood tree and maps obtained from the analysis of the European WNV-2 dataset. The tMRCA of tree root was estimated in 2002 (CI: 06/1999–08/2002). The earliest genome diverging from the root is an isolate obtained from a Goshawk in Hungary in 2004. The other genomes grouped into two main subclades, one containing the isolates from Greece since 2010 to 2022 (Greek subclade) with a tMRCA corresponding to 2007 (CI: 11/2006–02/2008), the other (Central European subclade) whose tMRCA dated 2006 (CI: 04/2004–04/2007) included sequences from different European countries such as Austria, Slovenia, Serbia, Germany and Italy. The majority (221/223, 99.1 %) of the Italian genomes, but one, formed a single highly supported (bootstrap value: 100 %) clade including also two Russian genomes collected in 2020 and two Spanish genomes collected in 2017 and 2020. The single isolate obtained in the Marche region in 2011, was at the outgroup of the clade, intermixed with other European genomes. The tMRCA of the pure Italian clade dated at the end of October 2009 (CI: 04/2009–06/2010). Two main Italian clades were observed: the eastern clade (clade A) having tMRCA in 2010 (CI: 07/2009–08/2010), which apparently went extinct in 2013–2014, and the western clade (clade B), dating in July 2012 (CI: 06/2012–10/2012) and persisting until today. Moreover, sequences sampled in Sardinia between 2018 and 2021 grouped in a single significant clade (clade C) dating in 2015 (CI: 05/2014–09/2016).

Finally, a single bird sample from Umbria collected in 2022 was included in the Greek subclade.

The phylogeographic analysis of the European isolates, reconstructed by ML method implemented in the Nextstrain package, suggested that the most probable origin of the virus was Hungary, followed by subsequent dispersion southward toward Serbia and Greece and westward toward Austria. Subsequently, from Austria, the virus was exported to several Central European countries, including Italy, from which the virus was exported to Spain and Russia. Two main entries can be observed in Italy from Austria: the first one reached Sardinia and then spread again to mainland Italy, representing the predominantly circulating clade in Italy even today, and another in the Marche, apparently without further spread in the country (see Supplementary Fig. S1).

A detailed visualization of the migration routes is available in the Nextstrain platform at the following link (Nextstrain / groups / Phylovir / WNV2EU334DEF).

### 3.4 Cluster analysis and continuous phylogeography of the WNV-2 Italian subset

The spread of WNV-2 in Italy was more deeply investigated by cluster analysis and continuous phylogeography using the Italian subset.

**Fig. 3:**
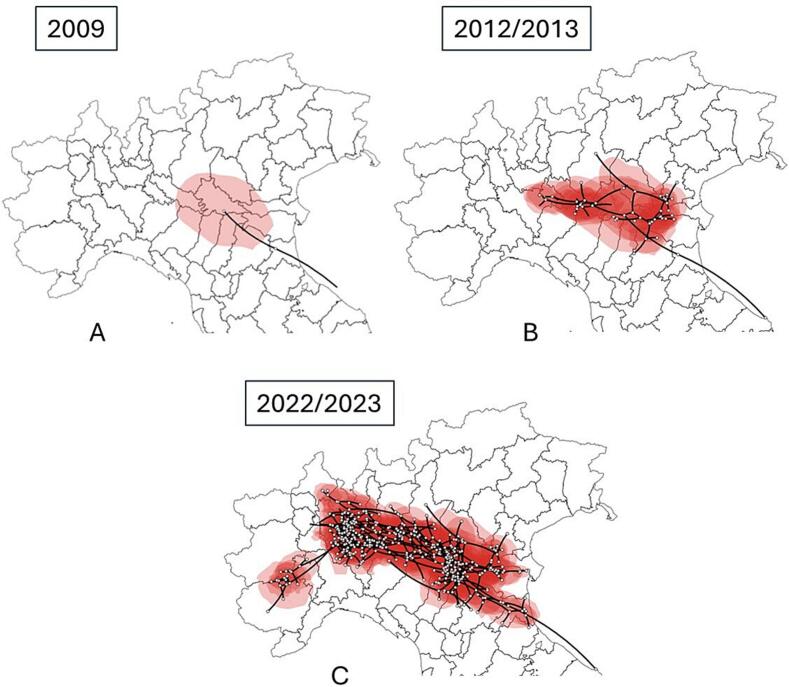
Spatio-temporal dynamics of the WNV-2 epidemic in Italy. Virus entry into Italy, 2009 (A), east-west spread between late 2012 and early 2013 (B). Western clade-only spread to Piedmont in the years 2022–2023 (C).

The cluster analysis (Fig. 3) showed that while the extinct subclade A formed only a single cluster, subclade B further bifurcated (in 2012–2013) into two main subclades (B1 and B2), the former including most strains isolated in the northwest (Piedmont), the latter those in the northeast (Veneto). Each of these subclades were further divided into distinct clusters (7 from B1 and 8 from B2), some of which in relation to sampling location (two for Piedmont, two for Lombardy and two for Veneto) and sampling time. In particular, the 2022 and 2023 samples formed clusters not observed before 2018 (Supplementary Table S4) with tMRCAs ranging from 2016 to 2021.

The largest clusters were B1.6 including 59 genomes collected in Piedmont and Lombardy during 2022 and 2023 seasons, B2.2 including 29 isolates from Emilia-Romagna, Veneto and Lombardy between 2013 and 2018, and B2.7 containing 20 isolates from Emilia-Romagna and Lombardy collected between 2021 and 2023.

The coordinates of the tree root estimated by continuous phylogeography were 44.50 “N and 11.93 “E, corresponding to an area located between the extreme eastern Po Valley, in the Emilia Romagna region, near Ravenna. The estimated tMRCA was 16.5 years ago (95 % HPD = 13.4–19.5), which suggests the entry of WNV-2 into Italy between late 2007 and early 2008 (Fig. 3A). Since 2011, the epidemic spread simultaneously eastwards and westwards (Fig. 3B). The eastern strain spread north-eastwards, reaching Ferrara, Rovigo and Padua in the first half of 2013 and currently appeared to be extinct. The western strain rapidly spread to an area between Cremona, Piacenza and Lodi and continued to expand westward and eastward since 2014. In particular, the epidemic gradually expanded westward reaching the Piedmont region in 2022 and 2023 (Figure 3C_ see animation in Dataverse UNIMI (https://doi.org/10.13130/RD_UNIMI/VIYNNE).

**Fig. 4:**
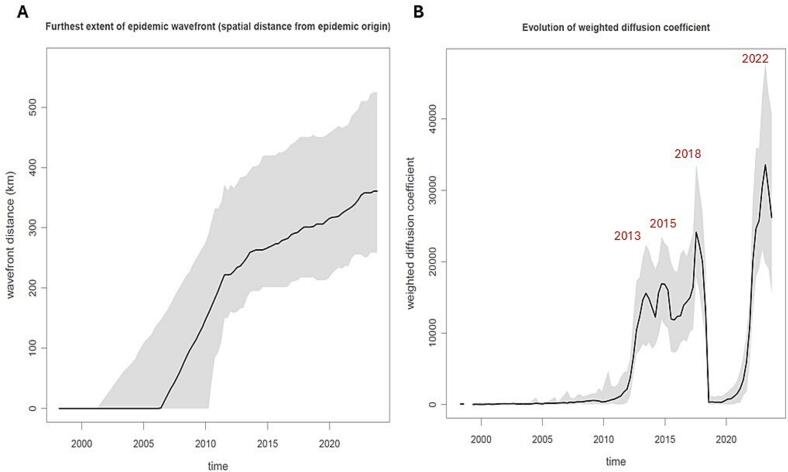
Extent of the epidemic wavefront (A) and the diffusion coefficient (B) plot of the WNV-2 italian epidemic.

The analysis with the R package “Seraphim” suggested a weighted branch dispersal velocity of 58.17 [95 %HPD 48.0–69.63] Km/year) (and mean branch dispersal velocity of 131.18 Km/year [95 %HPD 97.52–170.23]) and a median weighted diffusion coefficient of 661.55 Km2/year [95 %HPD 515.94–823.75]. Another key parameter is the extent of the epidemic wavefront (the maximum distance from the origin of the epidemic), which reached 350 km (850 km when the measure is based on patristic distance) in 2022–2023, 13 years later its estimated origin (Fig. 4 - panel A).

The diffusion coefficient reached several peaks during time: a first peak of the curve in 2013, followed by a smaller peak in 2015–2016 and highest peaks in 2018 and 2022 (Fig. 4 - panel B).

### 3.5 Nucleotide substitutions and selective pressure of newly characterized italian WNV-2 strains

**Fig. 5:**
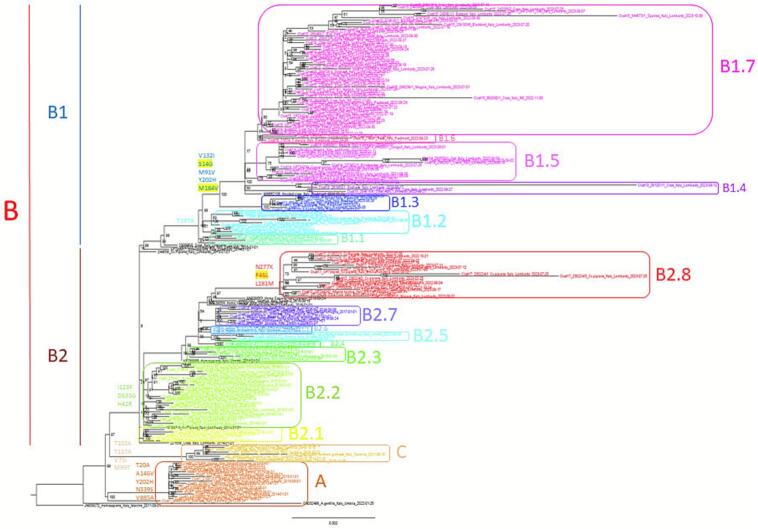
Maximum likelihood tree with significant subclusters coloured by Cluster Picker program. Non-synonymous mutations observed are indicated near each cluster and those under positive selective pressure are highlighted in yellow. (For interpretation of the references to colour in this figure legend, the reader is referred to the web version of this article.)

The mutational analysis revealed specific substitution patterns for each cluster (Fig. 5). Most of the mutations were synonymous except for those indicated below.

• Clade A (including mainly strains collected in 2013–2014 from Emilia Romagna and Veneto): preM-M (T20A); NS1 (A146V); NS5 (Y202H, N339S, V885A);
• Clade B (including sequences collected in Sardinia between 2018 and 2021): preM-M (T103A); E (T157A); NS2B (V75I, M99T).
• Clade C (including strains isolated from Lombardy, Piedmont, Emilia Romagna, Veneto in the period 2013–2023): preM-M E (A51T, Y155S, N277K,); NS1 (Y35H, I46V, I123F); NS2A (F46L, V132I) NS2B (V75I); NS3 (H249P, I450V); NS4B (S14G, L181M, M184V); NS5 (H42R, M91V, T197A, Y202H, D635G).

Analysis of the site-specific selection on the aligned protein-coding genes of the isolates obtained in 2022 and 2023 seasons (46 and 52, respectively), allowed the identification of a total of 18 sites under pervasive diversifying positive selection, summarized in Table 3. Six of them were present in more than 10 % of the samples.

**Table 3:**
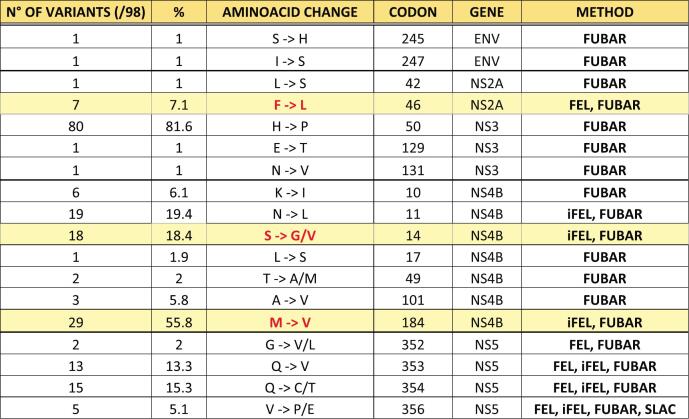
Codons under positive selective pressure in different genes, supported by at least one method, in the newly characterized sequences in 2022 and 2023.

Characteristic mutations of selected subclades are highlighted in yellow.

## 4 Discussion

WNV-2 related to the Hungarian isolates was first isolated in Italy in 2011, in environmental samples and also in sporadic human cases: a febrile patient in Ancona and in a patient with WNND in Sardinia [13,14,39]. Since then, it has been spreading throughout the country to become the prevalent lineage in Italy, which is one of the European countries with the highest number of reported cases [40].

The aim of this manuscript was to study the genomic epidemiology of West Nile virus in Italy in 2022 and 2023, in the light of previous epidemic waves. To this end, we characterized 98 whole genomes from animal samples (mosquito pools and avian organ homogenates) in Northern Italy during the 2022 and 2023 seasons by adding Italian and European available genomes collected in different times since 2004 to the present.

The newly sequenced whole genomes were obtained by two homemade protocols for the PCR amplification using either primer pools or specific primers. The new applied method for WGS allowed us to obtain sequences even from samples with a low viral load (≥30 Ct), a common condition in environmental isolates.

Present data confirmed results from our previous studies describing the evolutionary dynamics of WNV-2 in Italy. Since its introduction in 2011, Italian WNV-2 genomes formed a single clade segregating from other European isolates, further partitioned into two distinct subclades: clade A, previously circulating in northeastern Italy and apparently extinct by 2013, and clade B, prevalent in northwestern Italy, persisting to the present and spreading throughout the Po Valley [11,40]. More recently a group of isolates obtained in Sardinia in 2018–2021, formed a third clade, called in this paper C clade [41].

At the European level, previous studies [3,42] have reported WNV-2 introductions into Italy via Hungary in 2004, subsequent spread to central Europe, and further dispersal to Italy and the Balkans, which now appears to be an important center for the spread of the epidemic toward both north and west Europe (Germany, Czech Republic and Spain). The phylogeographic analysis carried out on the international dataset confirms these continental patterns but also provide additional resolution within Italy. Discrete phylogeography showed two separate introductions into Italy between 2009 and 2010; the first from Austria into Sardinia, which gave rise to the strain still currently circulating in Italy, and the second into Marche, which apparently did not further expand. From Sardinia, the virus spread to the Po Valley, and then it spread to northern Italy from 2012 onwards in a prevailing direction from east to west, forming two main subclades, already described in a previous article [11]: clade A, which became extinct between 2013 and 2014, and clade B, which has since become the prevalent clade still circulating mainly Westward reaching westernmost border of Italy in the last year.

The WNV-2 genomic epidemiology in Italy indicated that the isolates obtained in 2022 and 2023 were included in the clade B, including only few non-Italian isolates. This confirmed that WNV-2 is now enzootically circulating in our country, although sporadic introductions of different WNV-2 strains (e.g. the Umbria strain in a bird, closely related to the Greek strain) and different lineages (WNV-1) can sporadically occur.

In this ecological context, the detection of Usutu virus (USUV) in some mosquito pools collected in the same areas further supports the picture of a complex co-circulation of *Culex*-borne flaviviruses in Northern Italy. USUV shares with WNV a similar enzootic cycle involving ornithophilic mosquitoes and wild birds, and it is now considered endemic in several European countries, including Italy [43]. USUV has been rarely identified in humans, mainly in asymptomatic cases, but also in a small number of sever cases of neurological complications [44,45]. Although USUV was not the primary target of this study, its identification in our entomological samples underlines the added value of integrated genomic surveillance of mosquito-borne viruses, which can simultaneously capture the activity of WNV and other co-circulating arboviruses in a One Health perspective [46,47].

The clusters analysis has shown a partition of the main Italian B clade into two subclades B1 and B2, the former prevailing in the northwest and the latter in the northeast Italy. The two main subclades further divide into many clusters (only partially corresponding to those described in 2018 – [11]) grouping isolates obtained in successive years and in neighboring territories, thus suggesting persistence of locally circulating strains even over several seasons.

This East-West dichotomy in the spread routes of WNV has been visualised also by continuous phylogeography which reconstructs the viral dispersal in a two-dimensional space [48].

Continuous phylogeography has allowed the reconstruction of virus' spread routes with more precision, confirming the viral flow along the Po River's main tributaries, in areas with a high density of migratory birds and local avifauna. Our analyses confirm that the virus requires a period of enzootic circulation between reservoir animals (birds) and the mosquitoes that transmit it, before causing epidemics in humans [40].

Interestingly, the estimated dispersal velocity of the epidemic and of the diffusion coefficient shows values significantly lower (by about 100 times) than those highlighted for the WNV epidemic in the United States [49]. This probably reflects the different avian reservoirs involved in virus dispersion. The reconstruction of infection dispersal pathways suggests that local avifauna may be responsible for the more restricted intra-seasonal movements. In particular, European blackbirds (*Turdus merula*) might play a role in local transmission and short-distance spread of WNV, similarly to the role of the American robin (*Turdus migratorius*) in the United States, amplifying virus within local bird–mosquito cycles [50]. While they contribute substantially to virus maintenance and local amplification, their partially sedentary and particular migratory behavior in Europe indicates that they are less relevant for long-range introductions. These biological observations are consistent with the dispersal estimates obtained in our continuous phylogeography analyses, where localized virus movement predominates, punctuated by occasional longer-distance introductions likely mediated by other migratory species.

The analysis of the time-scaled evolution of the diffusion coefficient showed at least four increases: two initial peacks in 2013 and 2015, and two higher peacks in 2018 and 2022, suggesting a rapid spread of the virus. Although the estimated dispersal velocity in Italy is significantly lower than that reported for the WNV epidemic in the United States [49], these peaks reflect a spread over a wider geographic area compared to the baseline intra-seasonal dispersal, likely driven by local ecological factors such as avian reservoir distribution, vector abundance, and landscape connectivity.

The mutation analysis showed that the most relevant sub-clades were characterized by distinct mutational patterns, suggesting the co-circulation of different viral populations. Three mutations (F46L in NS2A, and S14G and M184V in NS4B) identified in subclade B2.8 and B1 were found under positive selective pressure in our dataset. To our knowledge, F46L, S14G, and M184V have not been previously reported in the literature with experimentally validated phenotypic effects. However, NS2A and NS4B proteins are known to play important roles in viral replication and immune evasion, and mutations in these proteins have been previously associated with changes in virulence or interferon antagonism [[51], [52], [53]]. Therefore, these sites represent candidates for further functional studies to investigate potential effects on viral fitness, neurotropism, or pathogenicity. Notably, these mutations were primarily detected in environmental samples (mosquito pools and birds), rather than in human-derived genomes. This pattern is consistent with the idea that the dominant selective pressures shaping WNV-2 evolution act within the enzootic cycle between ornithophilic mosquitoes and avian reservoirs, whereas humans, as dead-end hosts, are unlikely to contribute substantially to the fixation of adaptive variants. The evidence of positive selection acting on these sites, together with their circulation in nature, suggests that these mutations may confer a fitness advantage in the virus' natural transmission cycle.

The limitations of this study were mainly related to sampling and data availability. Most genomes were obtained from northern Italy which is overrepresented also respect to European and global genomes available, which may limit the reconstruction of WNV-2 phylodynamic and phylogeography. Moreover, our analyses mainly relied on environmental and animal samples and could not be systematically integrated with human data. As phylodynamic and phylogeographic inferences are sensitive to the density and representativeness of available sequences, future studies with more homogeneous sampling and additional genomic data from other Italian and European regions will be needed to refine these results.

## 5 Conclusions

Our results show how the spread of WNV-2 among humans reflects what happens, often a few months in advance, in the wild among avian reservoirs and/or vectors, highlighting the tight connections between human, animal and environmental health. This underscores the importance of integrated genomic surveillance of WNV and other Arboviruses at the human–animal–environment interface, in accordance with the One Health approach.

For this reason, the possibility of reconstructing the epidemiology of the virus in the environment by characterizing viral genomes from environmental samples, is a fundamental tool (in particular using continuous phylogeography), for knowing and understanding events that are difficult to measure and assess by the means of classical epidemiology and surveillance, as they occur in the wild. This is why we believe that enhanced environmental genomic surveillance can provide valuable information for public health authorities regarding the need and urgency of the measures to be adopted for the control of vector-borne infections.

## CRediT authorship contribution statement

**Carla della Ventura:** Writing – review & editing, Writing – original draft, Visualization, Validation, Supervision, Software, Methodology, Investigation, Formal analysis, Data curation, Conceptualization. **Maya Carrera:** Writing – review & editing, Methodology, Investigation, Data curation. **Francesco Defilippo:** Writing – review & editing, Data curation. **Davide Lelli:** Writing – review & editing, Data curation. **Chiara Nogarol:** Writing – review & editing, Data curation. **Maria Lucia Mandola:** Writing – review & editing, Data curation. **Alessia Lai:** Writing – review & editing, Visualization, Data curation. **Annalisa Bergna:** Writing – review & editing, Visualization. **Francesca Moroni:** Writing – review & editing, Validation. **Ana Moreno:** Writing – review & editing, Visualization, Data curation. **Gianguglielmo Zehender:** Writing – review & editing, Writing – original draft, Visualization, Validation, Supervision, Software, Methodology, Investigation, Funding acquisition, Formal analysis, Data curation, Conceptualization.

## Funding

This project has received funding from EU funding within the NextGeneration EU-MUR PNRR Extended Partnership initiative on Emerging Infectious Diseases (Project no. PE00000007, INF-ACT) and the “LINEA 2” project of the University of Milan.

## Declaration of competing interest

The authors declare that they have no known competing financial interests or personal relationships that could have appeared to influence the work reported in this manuscript.

## Data Availability

All sequences characterized in this study have been deposited in GenBank [c] (acc. n°: PX632813-PX633019) and will be released upon publication. The UNIMI and Nextstrain links will be fully functional and open-access at the time of publication.

## Appendix A Supplementary data

Supplementary Figure S1. Detailed migration route showed on Nextstrain platform. Two main entries in Italy from Austria in 2011: the first reached Sardinia and spread to mainland Italy, the second in the Marche. Image 1

Supplementary Table S1 Primer pool to amplify the complete genome of WNV-2. Image 2

Supplementary Table S2 Accession numbers of all sequences used for (a) Maximum Likelihood and Nextstrain analyses, (b) cluster identification, and (c) continuous phylogeography analyses. Sequences produced in this study are highlighted in red. Image 3

Supplementary Table S3 Summary of sequencing quality parameters for the samples included in the study. For each sample, the number or raw reads, sequencing depth (coverage), and base quality scores (Q20 and Q30) are reported. Image 4

Supplementary Table S4 List of the clusters observed in the Italian subset. For each cluster identified, the tMRCA, number of sequences, bootstrap and sampled region were indicated. Clusters with the newly characterized samples were highlighted in yellow. Image 5

Supplementary Material and Methods. Image 6
