# Supplementary material for: Genomic epidemiology and phylogeographic reconstruction of West Nile virus 2 in Italy from 2011 to 2023
Source: One Health. 2025 Dec 24;22:101310. doi: 10.1016/j.onehlt.2025.101310 (PMC12811532; doi:10.1016/j.onehlt.2025.101310)

Supplementary Figure S1. Detailed migration route showed on Nextstrain platform. Two main entries in Italy from Austria in 2011: the first reached Sardinia and spread to mainland Italy, the second in the Marche.


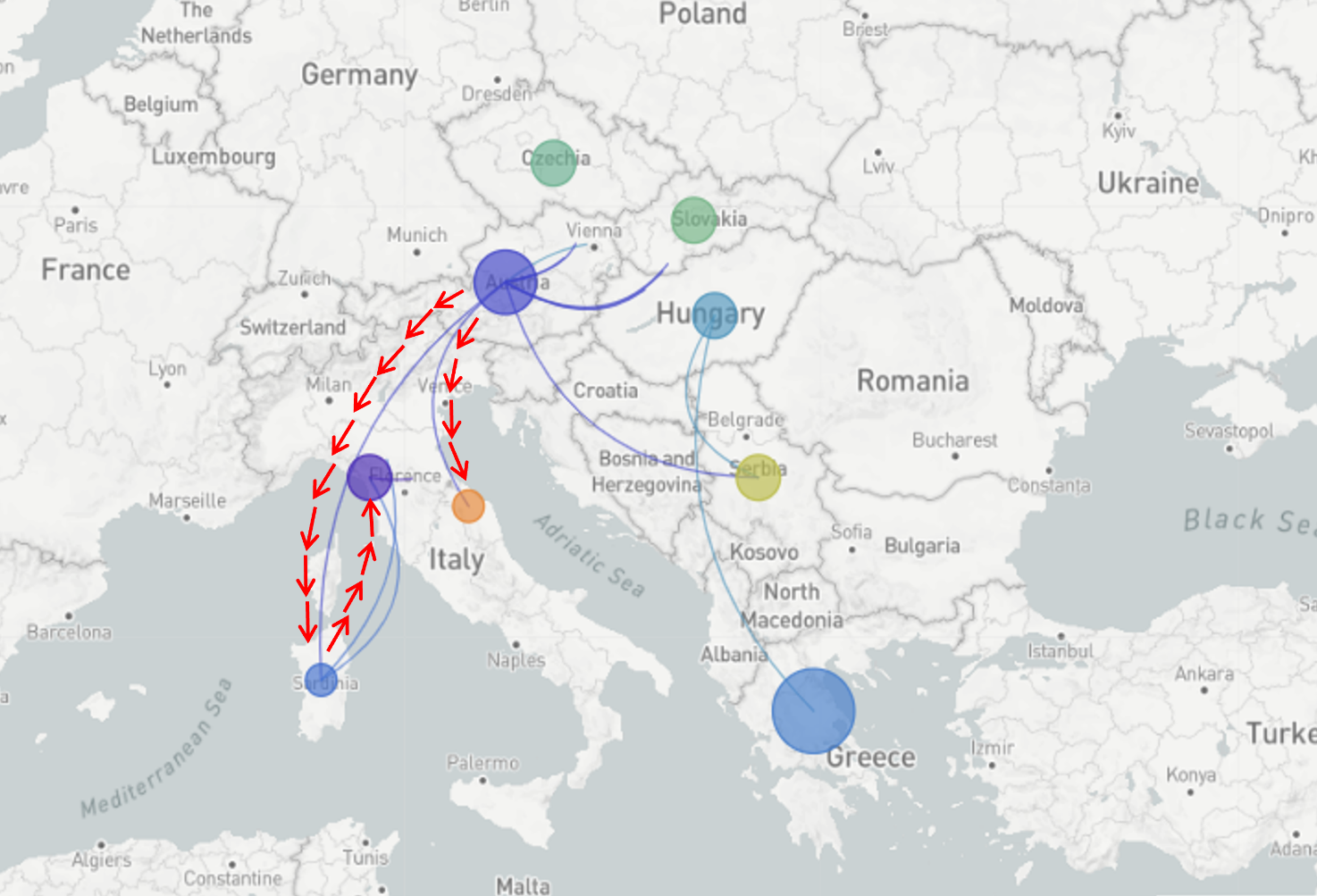

Supplement: Supplementary file 1 — Supplementary Figure S1. Detailed migration route showed on Nextstrain platform. Two main entries in Italy from Austria in 2011: the first reached Sardinia and spread to mainland Italy, the second in the Marche. [file mmc1.docx]
