# Supplementary material for: Genomic epidemiology and phylogeographic reconstruction of West Nile virus 2 in Italy from 2011 to 2023
Source: One Health. 2025 Dec 24;22:101310. doi: 10.1016/j.onehlt.2025.101310 (PMC12811532; doi:10.1016/j.onehlt.2025.101310)
Supplement: Supplementary Table S1 — Primer pool to amplify the complete genome of WNV-2. [file mmc2.docx]

Supplementary Table S1. Primer pool to amplify the complete genome of WNV-2.

| **Pool name** | **Sequence (5'→3')** | **Genome position (nt)** | **Amplified size (bp)** |
| --- | --- | --- | --- |
| WNV_poolOne | GATGTCTAAGAAACCAGGAGGGC | 96-118 | 401 |
| WNV_poolOne | ATCACTTTGCCCTGGAAGTTCG | 476-497 |  |
| WNV_poolOne | GCATTCCCGTCGAAGCAGAA | 720-739 | 401 |
| WNV_poolOne | TTGGCTGCTTCCATGTTCATCA | 1100-1121 |  |
| WNV_poolOne | GCCTGTACAACCAAAGCAACTG | 1324-1345 | 387 |
| WNV_poolOne | TTGTTTGGTGGCATGAGGTTCT | 1695-1711 |  |
| WNV_poolOne | CGGTGGTGTTGGAACTGCAATA | 1931-1952 | 410 |
| WNV_poolOne | AGGACATCCCTCCAAAGAGTGA | 2320-2341 |  |
| WNV_poolOne | TTCTACCCGGAGACACCACA | 2569-2588 | 409 |
| WNV_poolOne | CTCAGGAACATGCGAGTGCTT | 2958-2978 |  |
| WNV_poolOne | ATACCCATCACCTTGGCAGGA | 3196-3216 | 386 |
| WNV_poolOne | GGCCAAGAACACGACCAGAA | 3563-3582 |  |
| WNV_poolOne | TGAAGGCAAGGTGGACCAAC | 3809-3828 | 400 |
| WNV_poolOne | GTTGGGGTCGCAAGCCATTA | 4190-4209 |  |
| WNV_poolOne | GACATGTGGATTGAGAGGACGG | 4369-4390 | 419 |
| WNV_poolOne | CATGAGAGCAGCTCCCTTAGTG | 4767-4788 |  |
| WNV_poolOne | ACGTCAGGTTCCCCCATTGT | 5011-5030 | 417 |
| WNV_poolOne | GAGACATCAGCCTGTGTGTGAG | 5407-5428 |  |
| WNV_poolOne | AGCCTGGAACACTGGATATGAATG | 5640-5664 | 418 |
| WNV_poolOne | CATCCTCATTTGTGTGTCCTCCA | 6036-6058 |  |
| WNV_poolOne | CNGTCTGGCTCGCTTACAAAGT | 6242-6263 | 399 |
| WNV_poolOne | GCAATCAAAGCTATTGTCTGAAGGG | 6617-6641 |  |
| WNV_poolOne | GATTGTTTTGATTCCGGAGCCG | 6813-6834 | 398 |
| WNV_poolOne | AGCAAAAGAGCTGACACACCAA | 7190-7211 |  |
| WNV_poolOne | TGATCTTGGTGTCAATGGCCG | 7451-7471 | 386 |
| WNV_poolOne | CTCCAGTGATGTTTCCCTCTCTC | 7815-7837 |  |
| WNV_poolOne | ACCATGAAGAGCGGAGTCGA | 8056-8076 | 395 |
| WNV_poolOne | TTCCTCAAACTGGGGTCCCTT | 8431-8451 |  |
| WNV_poolOne | AGCTCCCTTGTGAATGGGGTA | 8641-8661 | 415 |
| WNV_poolOne | CCCATCATGTTGTAGATGCAGGT | 9034-9056 |  |
| WNV_poolOne | CAAAGCCTGGAGGAAAGATCTACG | 9260-9283 | 398 |
| WNV_poolOne | GCTCCTCGCCATTCTCAAACA | 9638-9658 |  |
| WNV_poolOne | TGGTATGACTGGCAGCAGGT | 9796-9815 | 390 |
| WNV_poolOne | CTGGTGTTTTGTCTTCCATCCAC | 10164-10186 |  |
| WNV_poolOne | TGAGGAGGTATGAAGACACCATTG | 10355-10378 | 414 |
| WNV_poolOne | TGCCTTTGTTAACCCAGTCCAC | 10748-10769 |  |

| WNV_poolTwo | ACAGCGGGCTTTACTATCTTGC | 418-439 | 402 |
| --- | --- | --- | --- |
| WNV_poolTwo | TCGTGGCTTTTGTGCTGTCC | 801-820 |  |
| WNV_poolTwo | TTGATCTGGTACTGGAAGGCGA | 1028-1049 | 389 |
| WNV_poolTwo | ATTCAACAGTCGTCGGGCCAT | 1397-1417 |  |
| WNV_poolTwo | ATGGATCTGAACCTGCCATGGA | 1621-1642 | 403 |
| WNV_poolTwo | CCAACAGGTGTGAGGTCATTCA | 2003-2024 |  |
| WNV_poolTwo | GATTTTGGGTCAGTTGGAGGGG | 2245-2261 | 418 |
| WNV_poolTwo | TGCTCGAGTCTGGAAACGGA | 2644-2663 |  |
| WNV_poolTwo | TTGTCATCGACGGTCCTGAGA | 2867-2887 | 411 |
| WNV_poolTwo | TCATCCCATGGACCTTGGTTCT | 3257-3278 |  |
| WNV_poolTwo | GAAAAGACCCTCGTGCAATCGA | 3493-3514 | 413 |
| WNV_poolTwo | CCATGACAAAACATTCTTGGCGT | 3884-3906 |  |
| WNV_poolTwo | GTTGGAGTTGGAAGCCTCATCA | 4066-4087 | 401 |
| WNV_poolTwo | TCCATCATCATCCAGCCTAACATC | 4444-4467 |  |
| WNV_poolTwo | TTGGCAGTTACCAAGCTGGAG | 4703-4723 | 406 |
| WNV_poolTwo | CACAATGGCGCTTATGTATGAACC | 5086-5109 |  |
| WNV_poolTwo | TACCAAACCTCAGCAGTGCAC | 5338-5358 | 397 |
| WNV_poolTwo | CAGAGGGCAATCTCATTTCCCA | 5714-5735 |  |
| WNV_poolTwo | GAAGGTGATGGAAGAGTCATCCTG | 5920-5943 | 412 |
| WNV_poolTwo | AGAATCGTGTTGGTTCGAGGTC | 6311-6332 |  |
| WNV_poolTwo | CACGATGTATGTGGTGGCAAC | 6543-6563 | 406 |
| WNV_poolTwo | CATTCTTGGTCTTGTCCAGCCA | 6928-6949 |  |
| WNV_poolTwo | CAATAAACGTCCAAGCCAGTGC | 7130-7151 | 400 |
| WNV_poolTwo | TGCTGTAGTCAGAATTCCAGCC | 7509-7530 |  |
| WNV_poolTwo | GACGAAGGAAGAATTTACCAGATACAGA | 7740-7767 | 404 |
| WNV_poolTwo | GCACTTGACGATGACTCTCCAA | 8123-8144 |  |
| WNV_poolTwo | TGAGATGTACTGGGTGAGCCA | 8334-8354 | 403 |
| WNV_poolTwo | CGAAAGGAGTGGTGTCTGTCAT | 8716-8737 |  |
| WNV_poolTwo | CAAAGTTTTGGGAGATGGTGGAC | 8970-8997 | 392 |
| WNV_poolTwo | TCCAGCAGTTCAAGAACCTTCG | 9340-9362 |  |
| WNV_poolTwo | ACCTACGCCCTGAACACCTT | 9508-9527 | 402 |
| WNV_poolTwo | CCCGTCCAATCAACTCGTCTT | 9890-9910 |  |
| WNV_poolTwo | GTGGATGGAAGACAAAACACCAG | 10164-10186 | 394 |
| WNV_poolTwo | CTTCCGGTGGCAGCATTAATCT | 10537-10558 |  |
