# Supplementary material for: Genomic epidemiology and phylogeographic reconstruction of West Nile virus 2 in Italy from 2011 to 2023
Source: One Health. 2025 Dec 24;22:101310. doi: 10.1016/j.onehlt.2025.101310 (PMC12811532; doi:10.1016/j.onehlt.2025.101310)
Supplement: Supplementary Table S2 — Accession numbers of all sequences used for (a) Maximum Likelihood and Nextstrain analyses, (b) cluster identification, and (c) continuous phylogeography analyses. Sequences produced in this study are highlighted in red. [file mmc3.docx]

Supplementary Table S2. Accession numbers of all sequences used for (a) Maximum Likelihood and Nextstrain analyses, (b) cluster identification, and (c) continuous phylogeography analyses. Sequences produced in this study are highlighted in red.

(a)

| **European dataset = 334 sequences (for ML and Nextstrain analyses)** | | | |
| --- | --- | --- | --- |
| **Acc. n°** | **Country** | **Location** | **Collection date** |
| DQ116961 | Hungary | Hungary | 2004-01-01 |
| HQ537483 | Greece | Greece | 2010-01-01 |
| KC407673 | Serbia | Serbia | 2012-01-01 |
| KF179639 | Greece | Greece | 2012-01-01 |
| KF179640 | Austria | Austria | 2008-01-01 |
| KC496015 | Hungary | Hungary | 2010-01-01 |
| KC496016 | Serbia | Serbia | 2010-01-01 |
| KM203860 | Czech.Republic | Czech.Republic | 2013-01-01 |
| KM203861 | Czech.Republic | Czech.Republic | 2013-01-01 |
| KM203862 | Czech.Republic | Czech.Republic | 2013-01-01 |
| KM203863 | Czech.Republic | Czech.Republic | 2013-01-01 |
| KM659876 | Austria | Austria | 2014-01-01 |
| KJ577738 | Greece | Greece | 2013-01-01 |
| KJ577739 | Greece | Greece | 2013-01-01 |
| KJ883341 | Greece | Greece | 2013-01-01 |
| KJ883342 | Greece | Greece | 2013-01-01 |
| KJ883343 | Greece | Greece | 2013-01-01 |
| KJ883344 | Greece | Greece | 2013-01-01 |
| KJ883345 | Greece | Greece | 2013-01-01 |
| KJ883346 | Greece | Greece | 2013-01-01 |
| KJ883347 | Greece | Greece | 2013-01-01 |
| KJ883348 | Greece | Greece | 2013-01-01 |
| KJ883349 | Greece | Greece | 2013-01-01 |
| KJ883350 | Greece | Greece | 2013-01-01 |
| KP109691 | Austria | Austria | 2014-01-01 |
| KP109692 | Austria | Austria | 2014-01-01 |
| KT359349.1 | Hungary | Hungary | 2014-01-01 |
| MF984337.1 | Austria | Austria | 2015-01-01 |
| MF984338.1 | Austria | Austria | 2015-01-01 |
| MF984339.1 | Austria | Austria | 2015-01-01 |
| MF984340.1 | Austria | Austria | 2015-01-01 |
| MF984341.1 | Austria | Austria | 2015-01-01 |
| MF984342.1 | Austria | Austria | 2015-01-01 |
| MF984343.1 | Austria | Austria | 2015-01-01 |
| MF984344.1 | Austria | Austria | 2015-01-01 |
| MF984345.1 | Austria | Austria | 2015-01-01 |
| MF984346.1 | Austria | Austria | 2016-01-01 |
| MF984347.1 | Austria | Austria | 2016-01-01 |
| MF984348.1 | Austria | Austria | 2016-01-01 |
| MF984349.1 | Austria | Austria | 2016-01-01 |
| MF984350.1 | Austria | Austria | 2016-01-01 |
| MF984351.1 | Austria | Austria | 2016-01-01 |
| MF984352.1 | Austria | Austria | 2016-01-01 |
| MH244510.1 | Slovakia | Slovakia | 2014-01-01 |
| MH244511.1 | Slovakia | Slovakia | 2013-01-01 |
| MH244512.1 | Slovakia | Slovakia | 2013-01-01 |
| MH244513.1 | Slovakia | Slovakia | 2013-01-01 |
| MH924836.1 | Germany | Germany | 2018-01-01 |
| MH986055.1 | Germany | Germany | 2018-01-01 |
| MH986056.1 | Germany | Germany | 2018-01-01 |
| JN858070 | Italy | Marche | 2011-09-01 |
| KF588365 | Italy | Veneto | 2013-08-01 |
| KF647248 | Italy | Veneto | 2013-08-01 |
| KF647249 | Italy | Veneto | 2013-08-01 |
| KF647250 | Italy | Veneto | 2013-08-01 |
| KF647251 | Italy | Veneto | 2013-08-01 |
| KF647252 | Italy | Veneto | 2013-08-01 |
| KF823805 | Italy | Veneto | 2013-08-01 |
| KF823806 | Italy | Lombardy | 2013-10-01 |
| KT207792 | Italy | Veneto | 2014-08-18 |
| KP789956 | Italy | Veneto | 2014-01-01 |
| KP789960 | Italy | Lombardy | 2014-01-01 |
| KP789959 | Italy | Lombardy | 2014-01-01 |
| KP789958 | Italy | Lombardy | 2014-01-01 |
| KP789957 | Italy | Lombardy | 2014-01-01 |
| KP789955 | Italy | Veneto | 2014-01-01 |
| KP789954 | Italy | Lombardy | 2014-01-01 |
| KP789953 | Italy | Lombardy | 2014-01-01 |
| MW142227 | Germany | Germany | 2020-08-01 |
| OM037670 | Spain | Catalonia | 2017-09-01 |
| OM037671 | Spain | Catalonia | 2020-09-01 |
| OP345105 | Russia | Volgograd | 2020-01-01 |
| OP345106 | Russia | Volgograd | 2020-01-01 |
| OP734242 | Italy | EmiliaRomagna | 2022-08-31 |
| OP734244 | Italy | Veneto | 2022-09-09 |
| OP850024 | Italy | EmiliaRomagna | 2022-08-04 |
| OP850025 | Italy | Piedmont | 2022-08-24 |
| OP850027 | Italy | Piedmont | 2021-08-10 |
| OP850028 | Italy | EmiliaRomagna | 2021-08-20 |
| OP850029 | Italy | EmiliaRomagna | 2021-10-24 |
| MN939557 | Italy | Veneto | 2018-08-24 |
| MN939558 | Italy | Veneto | 2016-08-24 |
| MN939559 | Italy | Veneto | 2016-08-24 |
| MN939560 | Italy | Veneto | 2016-08-24 |
| MN939561 | Italy | Veneto | 2018-08-24 |
| MN939562 | Italy | Veneto | 2018-08-24 |
| MN939563 | Italy | Veneto | 2018-08-24 |
| MN939564 | Italy | Veneto | 2016-08-24 |
| MW862073 | Italy | Sardinia | 2012-08-21 |
| MW862090 | Italy | Sardinia | 2018-07-24 |
| MW862091 | Italy | Sardinia | 2018-07-31 |
| MW862092 | Italy | Sardinia | 2018-08-13 |
| MW862093 | Italy | Sardinia | 2018-09-08 |
| MW862094 | Italy | Sardinia | 2019-07-15 |
| MW862095 | Italy | Sardinia | 2019-09-19 |
| MW862096 | Italy | Lombardy | 2018-08-31 |
| MW862097 | Italy | Sardinia | 2018-09-05 |
| MW862098 | Italy | Sardinia | 2018-10-13 |
| MW862099 | Italy | Sardinia | 2018-10-06 |
| MW862100 | Italy | Sardinia | 2018-10-02 |
| MW862101 | Italy | Sardinia | 2019-07-15 |
| MW862102 | Italy | Veneto | 2019-10-23 |
| MW862103 | Italy | Piedmont | 2019-09-12 |
| MW862104 | Italy | Piedmont | 2019-09-26 |
| MW862105 | Italy | Sardinia | 2019-10-10 |
| MW862106 | Italy | Piedmont | 2019-09-26 |
| MW862107 | Italy | Piedmont | 2019-08-26 |
| MW862108 | Italy | Piedmont | 2020-08-24 |
| ON032488 | Italy | Piedmont | 2020-09-07 |
| ON032489 | Italy | Piedmont | 2020-09-01 |
| ON032490 | Italy | Piedmont | 2021-08-19 |
| ON032491 | Italy | Lombardy | 2021-08-18 |
| ON032492 | Italy | EmiliaRomagna | 2021-08-26 |
| ON032493 | Italy | EmiliaRomagna | 2021-08-31 |
| ON032494 | Italy | Sardinia | 2021-09-10 |
| ON032495 | Italy | EmiliaRomagna | 2021-09-16 |
| ON032497 | Italy | EmiliaRomagna | 2021-08-04 |
| OP734238 | Italy | EmiliaRomagna | 2022-08-10 |
| OP734239 | Italy | EmiliaRomagna | 2022-08-10 |
| OQ053524 | Greece | Imathia | 2022-07-29 |
| OQ053525 | Greece | Kilkis | 2022-08-03 |
| OQ053521 | Greece | Thessaloniki | 2022-09-07 |
| OQ053522 | Greece | Thessaloniki | 2022-08-18 |
| OQ053519 | Greece | Thessaloniki | 2022-06-29 |
| OQ053526 | Greece | Serres | 2022-08-18 |
| OQ053527 | Greece | Chalkidiki | 2022-08-22 |
| OQ053523 | Greece | Thessaloniki | 2022-07-13 |
| OQ053520 | Greece | Pieria | 2022-08-31 |
| OQ053528 | Greece | Pieria | 2022-08-31 |
| OQ053529 | Greece | Pieria | 2022-08-29 |
| OQ053531 | Greece | Pella | 2022-08-24 |
| OQ053530 | Greece | Imathia | 2022-08-16 |
| OQ053515 | Greece | Chalkidiki | 2022-08-31 |
| OQ053536 | Greece | Serres | 2022-08-08 |
| OQ053535 | Greece | Thessaloniki | 2022-07-27 |
| OQ053532 | Greece | Thessaloniki | 2022-07-11 |
| OQ053533 | Greece | Imathia | 2022-08-24 |
| OL840899 | Greece | Thessaloniki | 2021-08-01 |
| OQ053518 | Greece | Larissa | 2022-07-26 |
| OQ053534 | Greece | Larissa | 2022-08-30 |
| OQ053516 | Greece | Larissa | 2022-08-19 |
| OQ053517 | Greece | Thessaloniki | 2022-08-16 |
| OL840896 | Greece | Serres | 2021-08-01 |
| OL840891 | Greece | Serres | 2020-08-01 |
| OL840897 | Greece | Imathia | 2021-08-01 |
| OL840898 | Greece | Thessaloniki | 2021-08-01 |
| OL840895 | Greece | Pella | 2021-08-01 |
| OL840894 | Greece | Thessaloniki | 2021-08-01 |
| OQ053539 | Greece | Pieria | 2022-07-18 |
| OQ053537 | Greece | Imathia | 2022-07-28 |
| OQ053538 | Greece | Imathia | 2022-08-10 |
| OL840887 | Greece | Serres | 2020-07-01 |
| OL840889 | Greece | Kilkis | 2020-08-01 |
| OL840883 | Greece | Serres | 2019-07-01 |
| OL840892 | Greece | Serres | 2020-08-01 |
| MT341472 | Bulgaria | Pleven | 2018-08-01 |
| MZ190464 | Kosovo | Kosovo | 2018-09-01 |
| OK239663 | Hungary | Nyiregyhaza | 2019-01-01 |
| OK239672 | Hungary | Tiszaluc | 2018-01-01 |
| OL840885 | Greece | Thessaloniki | 2019-08-01 |
| MN652880 | Greece | Thessaloniki | 2018-08-01 |
| MN480795 | Greece | Pella | 2018-08-01 |
| OL840884 | Greece | Kilkis | 2019-07-01 |
| MT341470 | Greece | Larissa | 2019-07-01 |
| MT341471 | Greece | Karditsa | 2019-07-01 |
| MN481591 | Greece | Rhodope | 2018-08-13 |
| KU206781 | Bulgaria | Sofia | 2015-08-27 |
| KT757319 | Serbia | Vojvodina | 2013-02-01 |
| MW751845 | Serbia | Opovo | 2018-07-12 |
| MH549209 | Greece | Argolida | 2017-07-01 |
| KY594040 | Greece | Thessaloniki | 2010-01-01 |
| MN481592 | Greece | Thessaloniki | 2012-08-02 |
| ON032498 | Italy | Umbria | 2022-01-25 |
| ON813233 | Italy | Piedmont | 2022-01-01 |
| OP734240 | Italy | Piedmont | 2022-08-18 |
| OP734241 | Italy | Piedmont | 2022-08-25 |
| OP734243 | Italy | EmiliaRomagna | 2022-09-02 |
| PX632959 | Italy | Lombardy | 2013-01-01 |
| PX632960 | Italy | Lombardy | 2013-01-01 |
| PX632961 | Italy | Lombardy | 2013-01-01 |
| PX632962 | Italy | EmiliaRomagna | 2013-01-01 |
| PX632963 | Italy | EmiliaRomagna | 2013-01-01 |
| PX632964 | Italy | EmiliaRomagna | 2013-01-01 |
| PX632965 | Italy | EmiliaRomagna | 2013-01-01 |
| PX632966 | Italy | Lombardy | 2013-01-01 |
| PX632967 | Italy | EmiliaRomagna | 2013-01-01 |
| PX632968 | Italy | Lombardy | 2013-01-01 |
| PX632969 | Italy | EmiliaRomagna | 2013-01-01 |
| PX632970 | Italy | Lombardy | 2014-01-01 |
| PX632971 | Italy | Lombardy | 2014-01-01 |
| PX632972 | Italy | Lombardy | 2014-01-01 |
| PX632973 | Italy | EmiliaRomagna | 2014-01-01 |
| PX632974 | Italy | EmiliaRomagna | 2014-01-01 |
| PX632975 | Italy | EmiliaRomagna | 2015-01-01 |
| PX632976 | Italy | EmiliaRomagna | 2015-01-01 |
| PX632977 | Italy | Lombardy | 2015-01-01 |
| PX632978 | Italy | Lombardy | 2015-01-01 |
| PX632979 | Italy | EmiliaRomagna | 2015-01-01 |
| PX632980 | Italy | EmiliaRomagna | 2015-01-01 |
| PX633002 | Italy | Lombardy | 2015-01-01 |
| PX633003 | Italy | Lombardy | 2015-01-01 |
| PX633004 | Italy | Lombardy | 2015-01-01 |
| PX633005 | Italy | Lombardy | 2015-01-01 |
| PX633006 | Italy | Lombardy | 2015-01-01 |
| PX632981 | Italy | EmiliaRomagna | 2015-01-01 |
| PX632982 | Italy | EmiliaRomagna | 2015-01-01 |
| PX632983 | Italy | EmiliaRomagna | 2015-01-01 |
| PX632984 | Italy | EmiliaRomagna | 2015-01-01 |
| PX632985 | Italy | EmiliaRomagna | 2015-01-01 |
| PX632986 | Italy | EmiliaRomagna | 2015-01-01 |
| PX633007 | Italy | EmiliaRomagna | 2016-01-01 |
| PX633008 | Italy | EmiliaRomagna | 2016-01-01 |
| PX633009 | Italy | EmiliaRomagna | 2017-01-01 |
| PX633010 | Italy | EmiliaRomagna | 2016-01-01 |
| PX633011 | Italy | EmiliaRomagna | 2016-01-01 |
| PX633012 | Italy | EmiliaRomagna | 2017-01-01 |
| PX633013 | Italy | EmiliaRomagna | 2017-01-01 |
| PX633014 | Italy | EmiliaRomagna | 2016-01-01 |
| PX633015 | Italy | EmiliaRomagna | 2016-01-01 |
| PX633016 | Italy | Lombardy | 2017-01-01 |
| PX633017 | Italy | EmiliaRomagna | 2016-01-01 |
| PX633018 | Italy | EmiliaRomagna | 2016-01-01 |
| PX633019 | Italy | EmiliaRomagna | 2016-01-01 |
| PX632987 | Italy | Lombardy | 2016-01-01 |
| PX632988 | Italy | Lombardy | 2016-01-01 |
| PX632999 | Italy | Lombardy | 2015-01-01 |
| PX633001 | Italy | Lombardy | 2016-01-01 |
| PX633000 | Italy | Lombardy | 2015-01-01 |
| PX632989 | Italy | EmiliaRomagna | 2016-01-01 |
| PX632990 | Italy | Lombardy | 2018-01-01 |
| PX632991 | Italy | Lombardy | 2018-01-01 |
| PX632992 | Italy | Lombardy | 2018-01-01 |
| PX632993 | Italy | Lombardy | 2018-01-01 |
| PX632994 | Italy | Lombardy | 2018-01-01 |
| PX632995 | Italy | Lombardy | 2018-01-01 |
| PX632996 | Italy | Lombardy | 2018-01-01 |
| PX632997 | Italy | Lombardy | 2018-01-01 |
| PX632998 | Italy | Lombardy | 2018-01-01 |
| PX632863 | Italy | Lombardy | 2022-07-05 |
| PX632864 | Italy | Lombardy | 2022-07-08 |
| PX632867 | Italy | Lombardy | 2022-07-19 |
| PX632865 | Italy | Lombardy | 2022-07-19 |
| PX632868 | Italy | Lombardy | 2022-07-19 |
| PX632869 | Italy | Lombardy | 2022-07-22 |
| PX632870 | Italy | Lombardy | 2022-07-22 |
| PX632871 | Italy | Lombardy | 2022-07-22 |
| PX632872 | Italy | Lombardy | 2022-08-17 |
| PX632873 | Italy | Lombardy | 2022-08-26 |
| PX632874 | Italy | Lombardy | 2022-08-26 |
| PX632875 | Italy | Lombardy | 2022-09-07 |
| PX632876 | Italy | Lombardy | 2022-09-27 |
| PX632877 | Italy | Lombardy | 2022-09-30 |
| PX632878 | Italy | Lombardy | 2022-10-06 |
| PX632866 | Italy | Lombardy | 2022-08-26 |
| PX632883 | Italy | Lombardy | 2022-09-27 |
| PX632879 | Italy | Lombardy | 2022-07-08 |
| PX632880 | Italy | Lombardy | 2022-07-19 |
| PX632881 | Italy | Lombardy | 2022-07-19 |
| PX632882 | Italy | Lombardy | 2022-08-26 |
| PX632952 | Italy | Lombardy | 2022-09-23 |
| PX632901 | Italy | Lombardy | 2022-09-27 |
| PX632884 | Italy | Lombardy | 2022-09-30 |
| PX632885 | Italy | Lombardy | 2022-09-30 |
| PX632886 | Italy | Lombardy | 2022-09-30 |
| PX632887 | Italy | Lombardy | 2022-10-21 |
| PX632888 | Italy | Lombardy | 2022-10-21 |
| PX632889 | Italy | Lombardy | 2022-11-30 |
| PX632890 | Italy | Lombardy | 2022-11-30 |
| PX632891 | Italy | Lombardy | 2022-11-30 |
| PX632892 | Italy | Lombardy | 2022-11-30 |
| PX632893 | Italy | Piedmont | 2022-11-28 |
| PX632894 | Italy | Piedmont | 2022-10-31 |
| PX632895 | Italy | Piedmont | 2022-08-10 |
| PX632896 | Italy | Piedmont | 2022-09-23 |
| PX632897 | Italy | Piedmont | 2022-10-13 |
| PX632898 | Italy | Piedmont | 2022-09-06 |
| PX632899 | Italy | Piedmont | 2022-10-31 |
| PX632900 | Italy | Piedmont | 2022-09-06 |
| PX632902 | Italy | Lombardy | 2023-07-04 |
| PX632903 | Italy | Lombardy | 2023-07-11 |
| PX632904 | Italy | Lombardy | 2023-07-11 |
| PX632905 | Italy | Lombardy | 2023-07-18 |
| PX632906 | Italy | Lombardy | 2023-07-20 |
| PX632907 | Italy | Lombardy | 2023-07-25 |
| PX632908 | Italy | Lombardy | 2023-07-25 |
| PX632909 | Italy | Lombardy | 2023-07-25 |
| PX632910 | Italy | Lombardy | 2023-07-25 |
| PX632911 | Italy | Lombardy | 2023-07-25 |
| PX632912 | Italy | Lombardy | 2023-07-05 |
| PX632913 | Italy | Lombardy | 2023-07-08 |
| PX632914 | Italy | Lombardy | 2023-07-12 |
| PX632915 | Italy | Lombardy | 2023-07-09 |
| PX632916 | Italy | Lombardy | 2023-07-10 |
| PX632917 | Italy | Lombardy | 2023-06-26 |
| PX632918 | Italy | Lombardy | 2023-07-23 |
| PX632919 | Italy | Lombardy | 2023-07-29 |
| PX632920 | Italy | Lombardy | 2023-07-26 |
| PX632921 | Italy | Lombardy | 2023-07-27 |
| PX632922 | Italy | Lombardy | 2023-08-04 |
| PX632923 | Italy | Lombardy | 2023-08-02 |
| PX632924 | Italy | Lombardy | 2023-07-24 |
| PX632925 | Italy | Lombardy | 2023-07-25 |
| PX632926 | Italy | Lombardy | 2023-07-26 |
| PX632927 | Italy | Lombardy | 2023-07-26 |
| PX632953 | Italy | Lombardy | 2023-07-20 |
| PX632928 | Italy | Lombardy | 2023-07-20 |
| PX632954 | Italy | Lombardy | 2023-08-03 |
| PX632929 | Italy | Lombardy | 2023-08-07 |
| PX632930 | Italy | Lombardy | 2023-08-01 |
| PX632931 | Italy | Lombardy | 2023-08-09 |
| PX632932 | Italy | Lombardy | 2023-08-06 |
| PX632955 | Italy | Lombardy | 2023-07-30 |
| PX632933 | Italy | Lombardy | 2023-08-18 |
| PX632934 | Italy | Lombardy | 2023-05-15 |
| PX632935 | Italy | Lombardy | 2023-08-08 |
| PX632936 | Italy | Lombardy | 2023-08-14 |
| PX632937 | Italy | Lombardy | 2023-09-01 |
| PX632938 | Italy | Lombardy | 2023-08-18 |
| PX632939 | Italy | Lombardy | 2023-08-13 |
| PX632940 | Italy | Lombardy | 2023-08-17 |
| PX632941 | Italy | Lombardy | 2023-08-24 |
| PX632942 | Italy | Lombardy | 2023-09-12 |
| PX632956 | Italy | Lombardy | 2023-09-02 |
| PX632943 | Italy | Lombardy | 2023-09-03 |
| PX632944 | Italy | Lombardy | 2023-09-02 |
| PX632945 | Italy | Lombardy | 2023-09-17 |
| PX632946 | Italy | Lombardy | 2023-07-01 |
| PX632957 | Italy | Lombardy | 2023-10-01 |
| PX632947 | Italy | Lombardy | 2023-10-30 |
| PX632948 | Italy | Piedmont | 2023-08-28 |
| PX632949 | Italy | Piedmont | 2023-09-06 |
| PX632950 | Italy | Piedmont | 2023-09-05 |
| PX632958 | Italy | Piedmont | 2023-08-28 |
| PX632951 | Italy | Piedmont | 2023-08-28 |

(b)

| **Italian subset = 226 sequences (Cluster identification)** | | | |
| --- | --- | --- | --- |
| **Acc. n°** | **Country** | **Location** | **Collection date** |
| JN858070 | Italy | Marche | 2011-09-01 |
| KF588365 | Italy | Veneto | 2013-08-01 |
| KF647248 | Italy | Veneto | 2013-08-01 |
| KF647249 | Italy | Veneto | 2013-08-01 |
| KF647250 | Italy | Veneto | 2013-08-01 |
| KF647251 | Italy | Veneto | 2013-08-01 |
| KF647252 | Italy | Veneto | 2013-08-01 |
| KF823805 | Italy | Veneto | 2013-08-01 |
| PX632959 | Italy | Lombardy | 2013-01-01 |
| PX632960 | Italy | Lombardy | 2013-01-01 |
| PX632961 | Italy | Lombardy | 2013-01-01 |
| KF823806 | Italy | Lombardy | 2013-10-01 |
| KT207792 | Italy | Veneto | 2014-08-18 |
| KP789956 | Italy | Veneto | 2014-01-01 |
| KP789960 | Italy | Lombardy | 2014-01-01 |
| KP789959 | Italy | Lombardy | 2014-01-01 |
| KP789958 | Italy | Lombardy | 2014-01-01 |
| KP789957 | Italy | Lombardy | 2014-01-01 |
| KP789955 | Italy | Veneto | 2014-01-01 |
| KP789954 | Italy | Lombardy | 2014-01-01 |
| KP789953 | Italy | Lombardy | 2014-01-01 |
| PX632962 | Italy | EmiliaRomagna | 2013-01-01 |
| PX632963 | Italy | EmiliaRomagna | 2013-01-01 |
| PX632964 | Italy | EmiliaRomagna | 2013-01-01 |
| PX632965 | Italy | EmiliaRomagna | 2013-01-01 |
| PX632966 | Italy | Lombardy | 2013-01-01 |
| PX632967 | Italy | EmiliaRomagna | 2013-01-01 |
| PX632968 | Italy | Lombardy | 2013-01-01 |
| PX632969 | Italy | EmiliaRomagna | 2013-01-01 |
| PX632970 | Italy | Lombardy | 2014-01-01 |
| PX632971 | Italy | Lombardy | 2014-01-01 |
| PX632972 | Italy | Lombardy | 2014-01-01 |
| PX632973 | Italy | EmiliaRomagna | 2014-01-01 |
| PX632974 | Italy | EmiliaRomagna | 2014-01-01 |
| PX632975 | Italy | EmiliaRomagna | 2015-01-01 |
| PX632976 | Italy | EmiliaRomagna | 2015-01-01 |
| PX632977 | Italy | Lombardy | 2015-01-01 |
| PX632978 | Italy | Lombardy | 2015-01-01 |
| PX632979 | Italy | EmiliaRomagna | 2015-01-01 |
| PX632980 | Italy | EmiliaRomagna | 2015-01-01 |
| PX633002 | Italy | Lombardy | 2015-01-01 |
| PX633003 | Italy | Lombardy | 2015-01-01 |
| PX633004 | Italy | Lombardy | 2015-01-01 |
| PX633005 | Italy | Lombardy | 2015-01-01 |
| PX633006 | Italy | Lombardy | 2015-01-01 |
| PX632981 | Italy | EmiliaRomagna | 2015-01-01 |
| PX632982 | Italy | EmiliaRomagna | 2015-01-01 |
| PX632983 | Italy | EmiliaRomagna | 2015-01-01 |
| PX632984 | Italy | EmiliaRomagna | 2015-01-01 |
| PX632985 | Italy | EmiliaRomagna | 2015-01-01 |
| PX632986 | Italy | EmiliaRomagna | 2015-01-01 |
| PX633007 | Italy | EmiliaRomagna | 2016-01-01 |
| PX633008 | Italy | EmiliaRomagna | 2016-01-01 |
| PX633009 | Italy | EmiliaRomagna | 2017-01-01 |
| PX633010 | Italy | EmiliaRomagna | 2016-01-01 |
| PX633011 | Italy | EmiliaRomagna | 2016-01-01 |
| PX633012 | Italy | EmiliaRomagna | 2017-01-01 |
| PX633013 | Italy | EmiliaRomagna | 2017-01-01 |
| PX633014 | Italy | EmiliaRomagna | 2016-01-01 |
| PX633015 | Italy | EmiliaRomagna | 2016-01-01 |
| PX633016 | Italy | Lombardy | 2017-01-01 |
| PX633017 | Italy | EmiliaRomagna | 2016-01-01 |
| PX633018 | Italy | EmiliaRomagna | 2016-01-01 |
| PX633019 | Italy | EmiliaRomagna | 2016-01-01 |
| PX632987 | Italy | Lombardy | 2016-01-01 |
| PX632988 | Italy | Lombardy | 2016-01-01 |
| PX632999 | Italy | Lombardy | 2015-01-01 |
| PX633001 | Italy | Lombardy | 2016-01-01 |
| PX633000 | Italy | Lombardy | 2015-01-01 |
| PX632989 | Italy | EmiliaRomagna | 2016-01-01 |
| PX632990 | Italy | Lombardy | 2018-01-01 |
| PX632991 | Italy | Lombardy | 2018-01-01 |
| PX632992 | Italy | Lombardy | 2018-01-01 |
| PX632993 | Italy | Lombardy | 2018-01-01 |
| PX632994 | Italy | Lombardy | 2018-01-01 |
| PX632995 | Italy | Lombardy | 2018-01-01 |
| PX632996 | Italy | Lombardy | 2018-01-01 |
| PX632997 | Italy | Lombardy | 2018-01-01 |
| PX632998 | Italy | Lombardy | 2018-01-01 |
| PX632863 | Italy | Lombardy | 2022-07-05 |
| PX632864 | Italy | Lombardy | 2022-07-08 |
| PX632867 | Italy | Lombardy | 2022-07-19 |
| PX632865 | Italy | Lombardy | 2022-07-19 |
| PX632868 | Italy | Lombardy | 2022-07-19 |
| PX632869 | Italy | Lombardy | 2022-07-22 |
| PX632870 | Italy | Lombardy | 2022-07-22 |
| PX632871 | Italy | Lombardy | 2022-07-22 |
| PX632872 | Italy | Lombardy | 2022-08-17 |
| PX632873 | Italy | Lombardy | 2022-08-26 |
| PX632874 | Italy | Lombardy | 2022-08-26 |
| PX632875 | Italy | Lombardy | 2022-09-07 |
| PX632876 | Italy | Lombardy | 2022-09-27 |
| PX632877 | Italy | Lombardy | 2022-09-30 |
| PX632878 | Italy | Lombardy | 2022-10-06 |
| PX632866 | Italy | Lombardy | 2022-08-26 |
| PX632901 | Italy | Lombardy | 2022-09-27 |
| OP734242 | Italy | EmiliaRomagna | 2022-08-31 |
| OP734244 | Italy | Veneto | 2022-09-09 |
| OP850024 | Italy | EmiliaRomagna | 2022-08-04 |
| OP850025 | Italy | Piedmont | 2022-08-24 |
| OP850027 | Italy | Piedmont | 2021-08-10 |
| OP850028 | Italy | EmiliaRomagna | 2021-08-20 |
| OP850029 | Italy | EmiliaRomagna | 2021-10-24 |
| MN939557 | Italy | Veneto | 2018-08-24 |
| MN939558 | Italy | Veneto | 2016-08-24 |
| MN939559 | Italy | Veneto | 2016-08-24 |
| MN939560 | Italy | Veneto | 2016-08-24 |
| MN939561 | Italy | Veneto | 2018-08-24 |
| MN939562 | Italy | Veneto | 2018-08-24 |
| MN939563 | Italy | Veneto | 2018-08-24 |
| MN939564 | Italy | Veneto | 2016-08-24 |
| MW862073 | Italy | Sardinia | 2012-08-21 |
| MW862090 | Italy | Sardinia | 2018-07-24 |
| MW862091 | Italy | Sardinia | 2018-07-31 |
| MW862092 | Italy | Sardinia | 2018-08-13 |
| MW862093 | Italy | Sardinia | 2018-09-08 |
| MW862094 | Italy | Sardinia | 2019-07-15 |
| MW862095 | Italy | Sardinia | 2019-09-19 |
| MW862096 | Italy | Lombardy | 2018-08-31 |
| MW862097 | Italy | Sardinia | 2018-09-05 |
| MW862098 | Italy | Sardinia | 2018-10-13 |
| MW862099 | Italy | Sardinia | 2018-10-06 |
| MW862100 | Italy | Sardinia | 2018-10-02 |
| MW862101 | Italy | Sardinia | 2019-07-15 |
| MW862102 | Italy | Veneto | 2019-10-23 |
| MW862103 | Italy | Piedmont | 2019-09-12 |
| MW862104 | Italy | Piedmont | 2019-09-26 |
| MW862105 | Italy | Sardinia | 2019-10-10 |
| MW862106 | Italy | Piedmont | 2019-09-26 |
| MW862107 | Italy | Piedmont | 2019-08-26 |
| MW862108 | Italy | Piedmont | 2020-08-24 |
| ON032488 | Italy | Piedmont | 2020-09-07 |
| ON032489 | Italy | Piedmont | 2020-09-01 |
| ON032490 | Italy | Piedmont | 2021-08-19 |
| ON032491 | Italy | Lombardy | 2021-08-18 |
| ON032492 | Italy | EmiliaRomagna | 2021-08-26 |
| ON032493 | Italy | EmiliaRomagna | 2021-08-31 |
| ON032494 | Italy | Sardinia | 2021-09-10 |
| ON032495 | Italy | EmiliaRomagna | 2021-09-16 |
| ON032497 | Italy | EmiliaRomagna | 2021-08-04 |
| OP734238 | Italy | EmiliaRomagna | 2022-08-10 |
| OP734239 | Italy | EmiliaRomagna | 2022-08-10 |
| PX632879 | Italy | Lombardy | 2022-07-08 |
| PX632880 | Italy | Lombardy | 2022-07-19 |
| PX632881 | Italy | Lombardy | 2022-07-19 |
| PX632882 | Italy | Lombardy | 2022-08-26 |
| PX632952 | Italy | Lombardy | 2022-09-23 |
| PX632901 | Italy | Lombardy | 2022-09-27 |
| PX632884 | Italy | Lombardy | 2022-09-30 |
| PX632885 | Italy | Lombardy | 2022-09-30 |
| PX632886 | Italy | Lombardy | 2022-09-30 |
| PX632887 | Italy | Lombardy | 2022-10-21 |
| PX632888 | Italy | Lombardy | 2022-10-21 |
| PX632889 | Italy | ND | 2022-11-30 |
| PX632890 | Italy | ND | 2022-11-30 |
| PX632891 | Italy | ND | 2022-11-30 |
| PX632892 | Italy | ND | 2022-11-30 |
| ON032498 | Italy | Umbria | 2022-01-25 |
| ON813233 | Italy | Piedmont | 2022-01-01 |
| OP734240 | Italy | Piedmont | 2022-08-18 |
| OP734241 | Italy | Piedmont | 2022-08-25 |
| OP734243 | Italy | EmiliaRomagna | 2022-09-02 |
| PX632893 | Italy | Piedmont | 2022-11-28 |
| PX632894 | Italy | Piedmont | 2022-10-31 |
| PX632895 | Italy | Piedmont | 2022-08-10 |
| PX632896 | Italy | Piedmont | 2022-09-23 |
| PX632897 | Italy | Piedmont | 2022-10-13 |
| PX632898 | Italy | Piedmont | 2022-09-06 |
| PX632899 | Italy | Piedmont | 2022-10-31 |
| PX632900 | Italy | Piedmont | 2022-09-06 |
| PX632902 | Italy | Lombardy | 2023-07-04 |
| PX632903 | Italy | Lombardy | 2023-07-11 |
| PX632904 | Italy | Lombardy | 2023-07-11 |
| PX632905 | Italy | Lombardy | 2023-07-18 |
| PX632906 | Italy | Lombardy | 2023-07-20 |
| PX632907 | Italy | Lombardy | 2023-07-25 |
| PX632908 | Italy | Lombardy | 2023-07-25 |
| PX632909 | Italy | Lombardy | 2023-07-25 |
| PX632910 | Italy | Lombardy | 2023-07-25 |
| PX632911 | Italy | Lombardy | 2023-07-25 |
| PX632912 | Italy | Lombardy | 2023-07-05 |
| PX632913 | Italy | Lombardy | 2023-07-08 |
| PX632914 | Italy | Lombardy | 2023-07-12 |
| PX632915 | Italy | Lombardy | 2023-07-09 |
| PX632916 | Italy | Lombardy | 2023-07-10 |
| PX632917 | Italy | Lombardy | 2023-06-26 |
| PX632918 | Italy | Lombardy | 2023-07-23 |
| PX632919 | Italy | Lombardy | 2023-07-29 |
| PX632920 | Italy | Lombardy | 2023-07-26 |
| PX632921 | Italy | Lombardy | 2023-07-27 |
| PX632922 | Italy | Lombardy | 2023-08-04 |
| PX632923 | Italy | Lombardy | 2023-08-02 |
| PX632924 | Italy | Lombardy | 2023-07-24 |
| PX632925 | Italy | Lombardy | 2023-07-25 |
| PX632926 | Italy | Lombardy | 2023-07-26 |
| PX632927 | Italy | Lombardy | 2023-07-26 |
| PX632953 | Italy | Lombardy | 2023-07-20 |
| PX632928 | Italy | Lombardy | 2023-07-20 |
| PX632954 | Italy | Lombardy | 2023-08-03 |
| PX632929 | Italy | Lombardy | 2023-08-07 |
| PX632930 | Italy | Lombardy | 2023-08-01 |
| PX632931 | Italy | Lombardy | 2023-08-09 |
| PX632932 | Italy | Lombardy | 2023-08-06 |
| PX632955 | Italy | Lombardy | 2023-07-30 |
| PX632933 | Italy | Lombardy | 2023-08-18 |
| PX632934 | Italy | Lombardy | 2023-05-15 |
| PX632935 | Italy | Lombardy | 2023-08-08 |
| PX632936 | Italy | Lombardy | 2023-08-14 |
| PX632937 | Italy | Lombardy | 2023-09-01 |
| PX632938 | Italy | Lombardy | 2023-08-18 |
| PX632939 | Italy | Lombardy | 2023-08-13 |
| PX632940 | Italy | Lombardy | 2023-08-17 |
| PX632941 | Italy | Lombardy | 2023-08-24 |
| PX632942 | Italy | Lombardy | 2023-09-12 |
| PX632956 | Italy | Lombardy | 2023-09-02 |
| PX632943 | Italy | Lombardy | 2023-09-03 |
| PX632944 | Italy | Lombardy | 2023-09-02 |
| PX632945 | Italy | Lombardy | 2023-09-17 |
| PX632946 | Italy | Lombardy | 2023-07-01 |
| PX632957 | Italy | Lombardy | 2023-10-01 |
| PX632947 | Italy | Lombardy | 2023-10-30 |
| PX632948 | Italy | Piedmont | 2023-08-28 |
| PX632949 | Italy | Piedmont | 2023-09-06 |
| PX632950 | Italy | Piedmont | 2023-09-05 |
| PX632958 | Italy | Piedmont | 2023-08-28 |
| PX632951 | Italy | Piedmont | 2023-08-28 |

(c)

| **Italian ENV-gene subset = 220 sequences (Continuous phylogeographic analysis)** | | | |
| --- | --- | --- | --- |
| **Acc. n°** | **Country** | **Location** | **Collection date** |
| JN858070 | Italy | Marche | 2011-09-01 |
| KF588365 | Italy | Veneto | 2013-08-01 |
| KF647248 | Italy | Veneto | 2013-08-01 |
| KF647249 | Italy | Veneto | 2013-08-01 |
| KF647250 | Italy | Veneto | 2013-08-01 |
| KF647251 | Italy | Veneto | 2013-08-01 |
| KF647252 | Italy | Veneto | 2013-08-01 |
| KF823805 | Italy | Veneto | 2013-08-01 |
| PX632959 | Italy | Lombardy | 2013-01-01 |
| PX632960 | Italy | Lombardy | 2013-01-01 |
| PX632961 | Italy | Lombardy | 2013-01-01 |
| KF823806 | Italy | Lombardy | 2013-10-01 |
| KT207792 | Italy | Veneto | 2014-08-18 |
| KP789956 | Italy | Veneto | 2014-01-01 |
| KP789960 | Italy | Lombardy | 2014-01-01 |
| KP789959 | Italy | Lombardy | 2014-01-01 |
| KP789958 | Italy | Lombardy | 2014-01-01 |
| KP789957 | Italy | Lombardy | 2014-01-01 |
| KP789955 | Italy | Veneto | 2014-01-01 |
| KP789954 | Italy | Lombardy | 2014-01-01 |
| KP789953 | Italy | Lombardy | 2014-01-01 |
| PX632962 | Italy | EmiliaRomagna | 2013-01-01 |
| PX632963 | Italy | EmiliaRomagna | 2013-01-01 |
| PX632964 | Italy | EmiliaRomagna | 2013-01-01 |
| PX632965 | Italy | EmiliaRomagna | 2013-01-01 |
| PX632966 | Italy | Lombardy | 2013-01-01 |
| PX632967 | Italy | EmiliaRomagna | 2013-01-01 |
| PX632968 | Italy | Lombardy | 2013-01-01 |
| PX632969 | Italy | EmiliaRomagna | 2013-01-01 |
| PX632970 | Italy | Lombardy | 2014-01-01 |
| PX632971 | Italy | Lombardy | 2014-01-01 |
| PX632972 | Italy | Lombardy | 2014-01-01 |
| PX632973 | Italy | EmiliaRomagna | 2014-01-01 |
| PX632974 | Italy | EmiliaRomagna | 2014-01-01 |
| PX632975 | Italy | EmiliaRomagna | 2015-01-01 |
| PX632976 | Italy | EmiliaRomagna | 2015-01-01 |
| PX632977 | Italy | Lombardy | 2015-01-01 |
| PX632978 | Italy | Lombardy | 2015-01-01 |
| PX632979 | Italy | EmiliaRomagna | 2015-01-01 |
| PX632980 | Italy | EmiliaRomagna | 2015-01-01 |
| PX633002 | Italy | Lombardy | 2015-01-01 |
| PX633003 | Italy | Lombardy | 2015-01-01 |
| PX633004 | Italy | Lombardy | 2015-01-01 |
| PX633005 | Italy | Lombardy | 2015-01-01 |
| PX633006 | Italy | Lombardy | 2015-01-01 |
| PX632981 | Italy | EmiliaRomagna | 2015-01-01 |
| PX632982 | Italy | EmiliaRomagna | 2015-01-01 |
| PX632983 | Italy | EmiliaRomagna | 2015-01-01 |
| PX632984 | Italy | EmiliaRomagna | 2015-01-01 |
| PX632985 | Italy | EmiliaRomagna | 2015-01-01 |
| PX632986 | Italy | EmiliaRomagna | 2015-01-01 |
| PX633007 | Italy | EmiliaRomagna | 2016-01-01 |
| PX633008 | Italy | EmiliaRomagna | 2016-01-01 |
| PX633009 | Italy | EmiliaRomagna | 2017-01-01 |
| PX633010 | Italy | EmiliaRomagna | 2016-01-01 |
| PX633011 | Italy | EmiliaRomagna | 2016-01-01 |
| PX633012 | Italy | EmiliaRomagna | 2017-01-01 |
| PX633013 | Italy | EmiliaRomagna | 2017-01-01 |
| PX633014 | Italy | EmiliaRomagna | 2016-01-01 |
| PX633015 | Italy | EmiliaRomagna | 2016-01-01 |
| PX633016 | Italy | Lombardy | 2017-01-01 |
| PX633017 | Italy | EmiliaRomagna | 2016-01-01 |
| PX633018 | Italy | EmiliaRomagna | 2016-01-01 |
| PX633019 | Italy | EmiliaRomagna | 2016-01-01 |
| PX632987 | Italy | Lombardy | 2016-01-01 |
| PX632988 | Italy | Lombardy | 2016-01-01 |
| PX632999 | Italy | Lombardy | 2015-01-01 |
| PX633001 | Italy | Lombardy | 2016-01-01 |
| PX633000 | Italy | Lombardy | 2015-01-01 |
| PX632989 | Italy | EmiliaRomagna | 2016-01-01 |
| PX632990 | Italy | Lombardy | 2018-01-01 |
| PX632991 | Italy | Lombardy | 2018-01-01 |
| PX632992 | Italy | Lombardy | 2018-01-01 |
| PX632993 | Italy | Lombardy | 2018-01-01 |
| PX632994 | Italy | Lombardy | 2018-01-01 |
| PX632995 | Italy | Lombardy | 2018-01-01 |
| PX632996 | Italy | Lombardy | 2018-01-01 |
| PX632997 | Italy | Lombardy | 2018-01-01 |
| PX632998 | Italy | Lombardy | 2018-01-01 |
| PX632863 | Italy | Lombardy | 2022-07-05 |
| PX632864 | Italy | Lombardy | 2022-07-08 |
| PX632867 | Italy | Lombardy | 2022-07-19 |
| PX632865 | Italy | Lombardy | 2022-07-19 |
| PX632868 | Italy | Lombardy | 2022-07-19 |
| PX632869 | Italy | Lombardy | 2022-07-22 |
| PX632870 | Italy | Lombardy | 2022-07-22 |
| PX632871 | Italy | Lombardy | 2022-07-22 |
| PX632872 | Italy | Lombardy | 2022-08-17 |
| PX632873 | Italy | Lombardy | 2022-08-26 |
| PX632874 | Italy | Lombardy | 2022-08-26 |
| PX632875 | Italy | Lombardy | 2022-09-07 |
| PX632876 | Italy | Lombardy | 2022-09-27 |
| PX632878 | Italy | Lombardy | 2022-10-06 |
| PX632866 | Italy | Lombardy | 2022-08-26 |
| PX632901 | Italy | Lombardy | 2022-09-27 |
| PX632879 | Italy | Lombardy | 2022-07-08 |
| PX632880 | Italy | Lombardy | 2022-07-19 |
| PX632881 | Italy | Lombardy | 2022-07-19 |
| PX632882 | Italy | Lombardy | 2022-08-26 |
| PX632952 | Italy | Lombardy | 2022-09-23 |
| PX632901 | Italy | Lombardy | 2022-09-27 |
| PX632887 | Italy | Lombardy | 2022-10-21 |
| PX632888 | Italy | Lombardy | 2022-10-21 |
| PX632889 | Italy | Lombardy | 2022-11-30 |
| PX632890 | Italy | Lombardy | 2022-11-30 |
| PX632891 | Italy | Lombardy | 2022-11-30 |
| PX632892 | Italy | Lombardy | 2022-11-30 |
| PX632893 | Italy | Piedmont | 2022-11-28 |
| PX632894 | Italy | Piedmont | 2022-10-31 |
| PX632895 | Italy | Piedmont | 2022-08-10 |
| PX632896 | Italy | Piedmont | 2022-09-23 |
| PX632897 | Italy | Piedmont | 2022-10-13 |
| PX632898 | Italy | Piedmont | 2022-09-06 |
| PX632899 | Italy | Piedmont | 2022-10-31 |
| PX632900 | Italy | Piedmont | 2022-09-06 |
| PX632902 | Italy | Lombardy | 2023-07-04 |
| PX632903 | Italy | Lombardy | 2023-07-11 |
| PX632904 | Italy | Lombardy | 2023-07-11 |
| PX632905 | Italy | Lombardy | 2023-07-18 |
| PX632906 | Italy | Lombardy | 2023-07-20 |
| PX632907 | Italy | Lombardy | 2023-07-25 |
| PX632908 | Italy | Lombardy | 2023-07-25 |
| PX632909 | Italy | Lombardy | 2023-07-25 |
| PX632910 | Italy | Lombardy | 2023-07-25 |
| PX632911 | Italy | Lombardy | 2023-07-25 |
| PX632912 | Italy | Lombardy | 2023-07-05 |
| PX632913 | Italy | Lombardy | 2023-07-08 |
| PX632914 | Italy | Lombardy | 2023-07-12 |
| PX632915 | Italy | Lombardy | 2023-07-09 |
| PX632916 | Italy | Lombardy | 2023-07-10 |
| PX632917 | Italy | Lombardy | 2023-06-26 |
| PX632918 | Italy | Lombardy | 2023-07-23 |
| PX632919 | Italy | Lombardy | 2023-07-29 |
| PX632920 | Italy | Lombardy | 2023-07-26 |
| PX632921 | Italy | Lombardy | 2023-07-27 |
| PX632922 | Italy | Lombardy | 2023-08-04 |
| PX632923 | Italy | Lombardy | 2023-08-02 |
| PX632924 | Italy | Lombardy | 2023-07-24 |
| PX632925 | Italy | Lombardy | 2023-07-25 |
| PX632926 | Italy | Lombardy | 2023-07-26 |
| PX632927 | Italy | Lombardy | 2023-07-26 |
| PX632953 | Italy | Lombardy | 2023-07-20 |
| PX632928 | Italy | Lombardy | 2023-07-20 |
| PX632954 | Italy | Lombardy | 2023-08-03 |
| PX632929 | Italy | Lombardy | 2023-08-07 |
| PX632930 | Italy | Lombardy | 2023-08-01 |
| PX632931 | Italy | Lombardy | 2023-08-09 |
| PX632932 | Italy | Lombardy | 2023-08-06 |
| PX632955 | Italy | Lombardy | 2023-07-30 |
| PX632933 | Italy | Lombardy | 2023-08-18 |
| PX632934 | Italy | Lombardy | 2023-05-15 |
| PX632935 | Italy | Lombardy | 2023-08-08 |
| PX632936 | Italy | Lombardy | 2023-08-14 |
| PX632937 | Italy | Lombardy | 2023-09-01 |
| PX632938 | Italy | Lombardy | 2023-08-18 |
| PX632939 | Italy | Lombardy | 2023-08-13 |
| PX632941 | Italy | Lombardy | 2023-08-24 |
| PX632942 | Italy | Lombardy | 2023-09-12 |
| PX632956 | Italy | Lombardy | 2023-09-02 |
| PX632943 | Italy | Lombardy | 2023-09-03 |
| PX632944 | Italy | Lombardy | 2023-09-02 |
| PX632945 | Italy | Lombardy | 2023-09-17 |
| PX632946 | Italy | Lombardy | 2023-07-01 |
| PX632957 | Italy | Lombardy | 2023-10-01 |
| PX632947 | Italy | Lombardy | 2023-10-30 |
| PX632948 | Italy | Piedmont | 2023-08-28 |
| PX632949 | Italy | Piedmont | 2023-09-06 |
| PX632950 | Italy | Piedmont | 2023-09-05 |
| PX632958 | Italy | Piedmont | 2023-08-28 |
| PX632951 | Italy | Piedmont | 2023-08-28 |
| PX632813 | Italy | Lombardy | 2015-01-01 |
| PX632814 | Italy | Lombardy | 2015-01-01 |
| PX632815 | Italy | Lombardy | 2016-01-01 |
| PX632816 | Italy | Lombardy | 2017-01-01 |
| PX632817 | Italy | Lombardy | 2016-01-01 |
| PX632818 | Italy | Lombardy | 2015-01-01 |
| PX632819 | Italy | Lombardy | 2017-01-01 |
| PX632820 | Italy | Lombardy | 2015-01-01 |
| PX632821 | Italy | EmiliaRomagna | 2017-01-01 |
| PX632822 | Italy | EmiliaRomagna | 2017-01-01 |
| PX632823 | Italy | EmiliaRomagna | 2017-01-01 |
| PX632824 | Italy | EmiliaRomagna | 2017-01-01 |
| PX632825 | Italy | EmiliaRomagna | 2017-01-01 |
| PX632826 | Italy | EmiliaRomagna | 2017-01-01 |
| PX632827 | Italy | EmiliaRomagna | 2017-01-01 |
| PX632828 | Italy | EmiliaRomagna | 2017-01-01 |
| PX632829 | Italy | EmiliaRomagna | 2016-01-01 |
| PX632830 | Italy | EmiliaRomagna | 2017-01-01 |
| PX632831 | Italy | EmiliaRomagna | 2017-01-01 |
| PX632832 | Italy | Lombardy | 2018-01-01 |
| PX632833 | Italy | Lombardy | 2018-01-01 |
| PX632834 | Italy | Lombardy | 2018-01-01 |
| PX632835 | Italy | Lombardy | 2018-01-01 |
| PX632836 | Italy | Lombardy | 2018-01-01 |
| PX632837 | Italy | Lombardy | 2018-01-01 |
| PX632838 | Italy | Lombardy | 2018-01-01 |
| PX632839 | Italy | Lombardy | 2018-01-01 |
| PX632840 | Italy | Lombardy | 2018-01-01 |
| PX632841 | Italy | Lombardy | 2018-01-01 |
| PX632842 | Italy | Lombardy | 2018-01-01 |
| PX632843 | Italy | Lombardy | 2018-01-01 |
| PX632844 | Italy | Lombardy | 2018-01-01 |
| PX632845 | Italy | Lombardy | 2018-01-01 |
| PX632846 | Italy | Lombardy | 2018-01-01 |
| PX632847 | Italy | Lombardy | 2018-01-01 |
| PX632848 | Italy | Lombardy | 2018-01-01 |
| PX632849 | Italy | Lombardy | 2018-01-01 |
| PX632850 | Italy | Lombardy | 2018-01-01 |
| PX632851 | Italy | Lombardy | 2018-01-01 |
| PX632852 | Italy | Lombardy | 2018-01-01 |
| PX632853 | Italy | Lombardy | 2018-01-01 |
| PX632854 | Italy | Lombardy | 2018-01-01 |
| PX632855 | Italy | Lombardy | 2018-01-01 |
| PX632856 | Italy | Lombardy | 2018-01-01 |
| PX632857 | Italy | Lombardy | 2018-01-01 |
| PX632858 | Italy | Lombardy | 2018-01-01 |
| PX632859 | Italy | Lombardy | 2018-01-01 |
| PX632860 | Italy | Lombardy | 2017-01-01 |
| PX632861 | Italy | EmiliaRomagna | 2017-01-01 |
| PX632862 | Italy | EmiliaRomagna | 2017-01-01 |
