# Supplementary material for: Genomic epidemiology and phylogeographic reconstruction of West Nile virus 2 in Italy from 2011 to 2023
Source: One Health. 2025 Dec 24;22:101310. doi: 10.1016/j.onehlt.2025.101310 (PMC12811532; doi:10.1016/j.onehlt.2025.101310)
Supplement: Supplementary Table S3 — Summary of sequencing quality parameters for the samples included in the study. For each sample, the number or raw reads, sequencing depth (coverage), and base quality scores (Q20 and Q30) are reported. [file mmc4.docx]

Supplementary Table S2. Summary of sequencing quality parameters for the samples included in the study. For each sample, the number of raw reads, sequencing depth (coverage), and base quality scores (Q20 and Q30) are reported.

| 2022 | | | | | |
| --- | --- | --- | --- | --- | --- |
| **Primer pool protocol** | | | | | |
| **ID sample** | **Reads** | **Mean Coverage (min - max)** | **Mean Confidence** | **Q-SCORE** | |
|  |  |  |  | **Q20 %** | **Q30 %** |
| 213522/1 | 282,894 | 4412,1 (0 - 51224) | 35,6 | 97,2 | 91,4 |
| 223840/2 | 468,356 | 6542,8 (0 - 71095) | 35,9 | 96,2 | 92,7 |
| 222978/2 | 1,081,798 | 14710,8 (0 - 116,838) | 35,7 | 97,3 | 91,7 |
| 230103/3 | 607,377 | 8048,2 (0 - 49018) | 35,5 | 95,9 | 91 |
| 230103/5 | 514,274 | 6778,3 (0 - 16300) | 35,5 | 97 | 91,1 |
| 230103/6 | 338,597 | 5044,8 (0 - 32081) | 35,4 | 94,4 | 90,4 |
| 230111/2 | 1,185,527 | 1670,1 (0 - 169678) | 35,8 | 97,4 | 92,2 |
| 230117/2 | 727,722 | 9265,2 (0 - 21065) | 35,6 | 97,2 | 91,5 |
| 235169/1 | 586,278 | 7455,7 (0 - 16134) | 35,6 | 97,2 | 91,6 |
| 235169/3 | 443,26 | 5713,1 (0 - 11749) | 35,5 | 97,1 | 91,2 |
| 235169/4 | 369,491 | 4850,7 (0 - 10380) | 35,6 | 97,2 | 91,4 |
| 258815/5 | 334,042 | 3797,9 (0 - 9382) | 35,6 | 97,3 | 91,7 |
| 273738 | 389,608 | 5292,6 (0 - 57570) | 36 | 95,2 | 92,9 |
| 268468/1 | 463,712 | 5581,6 (0 - 13762) | 35,6 | 97,2 | 91,6 |
| 268468/7 | 945,91 | 13903,6 (0 - 168255) | 35,8 | 97,3 | 92 |
| 268475/6 | 239,418 | 2746,5 (0 - 7009) | 35,8 | 97,7 | 92,5 |
| 284740 | 193,973 | 2503,7 (0 - 5406) | 35,7 | 97,5 | 92,1 |
| 297996/18 | 577,176 | 8057,5 (0 - 56836) | 35,5 | 95,7 | 91 |
| 305987 | 290,904 | 3645,8 (0 - 9201) | 35,6 | 97,2 | 91,5 |
| 284616/6 | 699,756 | 9593,5 (0 - 49138) | 35,6 | 95,1 | 91 |
| 314750/5 | 324,444 | 4649,5 (0 - 45950) | 35,9 | 95,1 | 92,1 |
| 314750/8 | 570,351 | 8443,9 (0 - 85519) | 35,6 | 94,9 | 90,9 |
| 314750/16 | 60,119 | 731,7 (0 - 4647) | 35,8 | 95 | 91,8 |
| 314750/19 | 589,985 | 6350,7 (0 - 17671) | 35,8 | 97,6 | 92,4 |
| 316654 | 290,562 | 3768,4 (0 - 10461) | 35,6 | 97,2 | 91,4 |
| 335042 | 626,088 | 8850,1 (0 - 70150) | 35,5 | 95,2 | 90,9 |
| 337721/1 | 515,093 | 6721,5 (0 - 51259) | 35,9 | 96,4 | 92,4 |
| 216582/3 | 531,89 | 6516 (0 - 12899) | 36,1 | 94,3 | 92,9 |
| 250643/1 | 420,803 | 5477 (0 - 11908) | 35,6 | 97,2 | 91,6 |
| 254006/1 | 174,434 | 2271,2 (0 - 5775) | 35,4 | 96,8 | 90,5 |
| 382830/1 | 294,308 | 3991 (0 - 38834) | 35,9 | 95,5 | 92,5 |
| 354123/1 | 495,541 | 6592,7 (0 - 68313) | 35,9 | 95,8 | 92,5 |
| 1683 | 1,666,537 | 18622,6 (0 - 44749) | 36,3 | 94,8 | 93,8 |
| 1663/64 | 1,395,297 | 16032 (0 - 34427) | 36,5 | 95,6 | 94,6 |
| 1679 | 1,058,170 | 12632 (0 - 26257) | 36,3 | 95 | 93,9 |
| 1687 | 1,867,154 | 24415,9 (0 - 157485) | 36,1 | 94,2 | 93 |
| 1682 | 1,744,472 | 20618,2 (0 - 56242) | 36,3 | 95,1 | 93,9 |
| 1684 | 2,908,804 | 38888,2 (0 - 83125) | 36,2 | 94,4 | 93,3 |
| 1686 | 3,280,530 | 38626,9 (0 - 106309) | 36,3 | 94,8 | 93,7 |
| 1665 | 2,408,604 | 28707,5 (0 - 59483) | 36,3 | 95,1 | 93,9 |
| 1685 | 2,928,185 | 34342,7 (0 - 95341) | 36,4 | 95 | 94 |
| **Specific primers protocol** | | | | | |
| **ID sample** | **Reads** | **Mean Coverage (min - max)** | **Mean Confidence** | **Q-SCORE** | |
|  |  |  |  | **Q20 %** | **Q30 %** |
| **WNV_50** | 424,081 | 5812 (0 - 17845) | 36,2 | 94,5 | 93 |
| **WNV_53** | 412,704 | 5561,1 (0 - 26470) | 36,3 | 94,9 | 93,6 |
| **WNV_55** | 821,372 | 10381,4 (0 - 24889) | 36,1 | 94,4 | 93 |
|  |  |  |  |  |  |
|  |  |  |  |  |  |
|  |  |  |  |  |  |
| 2023 | | | | | |
| **Primer pool protocol** | | | | | |
| **ID sample** | **Reads** | **Mean Coverage (min - max)** | **Mean Confidence** | **Q-SCORE** | |
|  |  |  |  | **Q20 %** | **Q30 %** |
| 206744/2 | 578,858 | 7494.9 (0 - 15205) | 37.3 | 97.6 | 96.9 |
| 215424/4 | 458,677 | 5694.9 (0 - 19684) | 37.2 | 97.2 | 96.4 |
| 215419/2 | 202,889 | 2416.4 (0 - 5372) | 37.4 | 97.9 | 97.3 |
| 221998/1 | 7,95 | 96.6 (0 - 261) | 37.2 | 97.3 | 96.7 |
| 226265/3 | 412,588 | 5210.4 (0 - 11576) | 37.4 | 97.8 | 97.2 |
| 230377/2 | 777,948 | 9974.0 (0 - 18993) | 37 | 96.8 | 96 |
| 230224/1 | 479,739 | 6154.5 (0 - 16617) | 37.3 | 97.6 | 97 |
| 230224/2 | 753,842 | 9825.3 (0 - 21098) | 37.1 | 97 | 96.2 |
| 230224/3 | 332,891 | 4278.4 (0 - 14710) | 37.3 | 97.6 | 97 |
| 230224/4 | 345,177 | 4528.2 (0 - 11403) | 37.3 | 97.6 | 97 |
| 220260/1 | 484,499 | 5896.3 (0 - 12805) | 37.4 | 97.9 | 97.3 |
| 220272/1 | 347,86 | 4245.4 (0 - 9344) | 37.4 | 97.8 | 97.2 |
| 221085/2 | 194,973 | 2548.7 (0 - 8443) | 37.3 | 97.5 | 96.8 |
| 230861/1 | 910,102 | 12079.0 (0 - 21953) | 37.4 | 97.8 | 97.2 |
| 236346/1 | 882,133 | 11292.0 (0 - 28404) | 37.3 | 97.4 | 96.8 |
| 239463/1 | 496,35 | 6569.0 (0 - 13705) | 36.8 | 96 | 95 |
| 239469/1 | 813,858 | 10314.4 (0 - 23949) | 37.4 | 97.9 | 97.3 |
| 239474/1 | 139,956 | 1678.8 (0 - 5738) | 37.3 | 97.5 | 96.8 |
| 237992/1 | 185,637 | 2475.3 (0 - 5350) | 36.6 | 95.6 | 94.5 |
| 242433/1 | 380,55 | 4652.0 (0 - 10846) | 37.3 | 97.6 | 97 |
| 247194/1 | 317,608 | 3966.2 (0 - 7967) | 37.3 | 97.8 | 97.1 |
| 239061/2 | 63,783 | 990.7 (0 - 2600) | 37.3 | 97.5 | 96.7 |
| 243335/1 | 636,37 | 8150.2 (0 - 18626) | 37.4 | 97.7 | 97.1 |
| 243335/2 | 423,148 | 5632.2 (0 - 17586) | 37.2 | 97.4 | 96.7 |
| 243386/1 | 512,781 | 6416.0 (0 - 15477) | 37.3 | 97.7 | 97 |
| 243401/1 | 455,423 | 5641.6 (0 - 14041) | 37.3 | 97.7 | 97.1 |
| 239183/8 | 99,409 | 1394.5 (0 - 6999) | 37.4 | 97.7 | 97.1 |
| 239130/2 | 664,999 | 8590.5 (0 - 18679) | 37.3 | 97.6 | 96.9 |
| 247000/1 | 520,236 | 6454.3 (0 - 13091) | 37.2 | 97.4 | 96.7 |
| 247213/1 | 441,368 | 6034.3 (0 - 17860) | 37.4 | 97.8 | 97.1 |
| 244280/1 | 189,012 | 2294.5 (0 - 5197) | 37.4 | 97.8 | 97.2 |
| 249388/5 | 48,126 | 560.0 (0 - 1426) | 37.1 | 97.2 | 96.5 |
| 251594/1 | 325,472 | 4286.7 (0 - 12544) | 37.3 | 97.8 | 97.2 |
| 251447/1 | 539,3 | 7263.2 (0 - 16188) | 37.4 | 98 | 97.4 |
| 254796/4 | 325,703 | 4011.6 (0 - 8551) | 37.3 | 97.5 | 96.9 |
| 255298/1 | 364,28 | 4335.0 (0 - 9754) | 37.4 | 97.8 | 97.3 |
| 255348/1 | 306,348 | 3838.7 (0 - 8341) | 37.1 | 97.1 | 96.4 |
| 256225/1 | 604,006 | 7970.1 (0 - 19926) | 37.4 | 97.8 | 97.2 |
| 269576/1 | 429,269 | 5919.0 (0 - 11900) | 37.3 | 97.6 | 97 |
| 259250/1 | 295,352 | 4122.9 (0 - 12144) | 37.3 | 97.4 | 96.7 |
| 264195/1 | 732,281 | 9175.6 (0 - 18449) | 37.4 | 97.7 | 97.1 |
| 261963/1 | 667,047 | 8382.2 (0 - 16657) | 37.3 | 97.5 | 96.9 |
| 274846/1 | 517,902 | 6304.0 (0 - 13473) | 37.4 | 97.8 | 97.2 |
| 281201/1 | 22,193 | 286.3 (0 - 1289) | 37.5 | 98.1 | 97.5 |
| 274918/1 | 109,537 | 1328.6 (0 - 4140) | 37.4 | 97.8 | 97.2 |
| 283570/1 | 136,686 | 1796.5 (0 - 4688) | 37.1 | 97.1 | 96.4 |
| 283564/1 | 248,305 | 3215.2 (0 - 7168) | 37.3 | 97.5 | 96.8 |
| 296265/1 | 147,679 | 1778.0 (0 - 4227) | 37.3 | 97.5 | 96.8 |
| 296239/1 | 357,542 | 4440.2 (0 - 11009) | 37.2 | 97.3 | 96.6 |
| 306868/1 | 387,357 | 5019.8 (0 - 11254) | 37.1 | 96.9 | 96.2 |
| 344573/1 | 359,264 | 4873.0 (0 - 16751) | 37.3 | 97.6 | 97 |
| 344576/1 | 335,107 | 4061.7 (0 - 14330) | 37.4 | 97.9 | 97.3 |
| 75264 | 505,297 | 6323.5 (0 - 16019) | 36.7 | 96.3 | 95.3 |
| 77490/7 | 353,018 | 4380.3 (0 - 16061) | 36.6 | 95.8 | 94.7 |
| 77311/1 | 759,968 | 9377.1 (0 - 21207) | 36.7 | 96.1 | 95.1 |
| 75257 | 319,903 | 4048.4 (0 - 12261) | 36.6 | 95.8 | 94.8 |
| 75255 | 1,073,925 | 13640.6 (0 - 32123) | 35.8 | 93.2 | 91.7 |
