# Supplementary material for: Genomic epidemiology and phylogeographic reconstruction of West Nile virus 2 in Italy from 2011 to 2023
Source: One Health. 2025 Dec 24;22:101310. doi: 10.1016/j.onehlt.2025.101310 (PMC12811532; doi:10.1016/j.onehlt.2025.101310)
Supplement: Supplementary Table S4 — List of the clusters observed in the Italian subset. For each cluster identified, the tMRCA, number of sequences, bootstrap and sampled region were indicated. Clusters with the newly characterized samples were highlighted in yellow. [file mmc5.docx]

Supplementary Table 3. List of the clusters observed in the Italian subset. For each cluster identified, the tMRCA, number of sequences, bootstrap and sampled region were indicated. Cluster with the newly characterized samples were highlighted in yellow.
