# Supplementary material for: Genomic epidemiology and phylogeographic reconstruction of West Nile virus 2 in Italy from 2011 to 2023
Source: One Health. 2025 Dec 24;22:101310. doi: 10.1016/j.onehlt.2025.101310 (PMC12811532; doi:10.1016/j.onehlt.2025.101310)
Supplement: Supplementary file 6 — Supplementary Material and Methods. [file mmc6.docx]

**Supplementary Materials and Method**

**Sample processing**

**Nucleic Acid Extraction and RNA quantification**

Reagents

QIAMP viral RNA mini-Kit (Qiagen GmbH, Germany) for 250 viral RNA extractions, consisted of:

- QIAamp Mini Spin Columns
- Collection Tubes (2 mL)
- Buffer AVL, ready to use
- Buffer AW1, diluited by adding 130 mL 96-100% ehanol to each bottle at the first use
- Buffer AW2, diluited by adding 160 mL 96-100% ehanol to each bottle at the first use
- Buffer AVE, ready to use
- Carrier RNA (poly A), resuspended by adding 1500 uL of buffer AVE to each tube at the first use

Equipment:

- Ethanol (96–100%)
- 1.5 mL microcentrifuge tubes
- Sterile, RNase-free pipette tips
- Microcentrifuge

Protocol

1. Pipet 560 µL prepared Buffer AVL and 5.6 uL of carrier RNA into a 2 mL microcentrifuge tube.
2. Add 140 µL of the sample to the Buffer AVL–carrier RNA in the microcentrifuge tube. Mix by pulse-vortexing for 15 sec.
3. Incubate at room temperature for 10 min.
4. Briefly centrifuge the tube to remove drops from the inside of the lid.
5. Add 560 µL ethanol (96–100%) to the sample, and mix by pulse-vortexing for 15 s. After mixing, briefly centrifuge the tube to remove drops from inside the lid.
6. Apply 630 µL of the solution from step 5 to the QIAamp Mini column (in a 2 mL collection tube) without wetting the rim. Close the cap, and centrifuge at 8000 rpm for 1 min. Place the QIAamp Mini column into a clean 2 mL collection tube, and discard the tube containing the filtrate.
7. Open the QIAamp Mini column, and repeat step 6 until all of the lysate has been loaded into the spin column.
8. Add 500 µL Buffer AW1. Close the cap, and centrifuge at 8000 rpm for 1 min. Place the QIAamp Mini column in a clean 2 mL collection tube, and discard the tube containing the filtrate.
9. Add 500 µL Buffer AW2. Close the cap and centrifuge at 8000 rpm for 1 min.
10. : Place the QIAamp Mini column in a new 2 mL collection tube, and discard the old collection tube with the filtrate. Centrifuge at full speed (14000 rpm) for 3 min.
11. Place the QIAamp Mini column in a clean 1.5 mL microcentrifuge tube. Discard the old collection tube containing the filtrate. Open the QIAamp Mini column and add 50 µL RNAse-free water water heated to a temperature of 56 ° C. Close the cap, and incubate at room temperature for 5 min.
12. Centrifuge at 8000 rpmfor 1 min. Discard the QIAmp Mini column and store the microcentrifuge tube with the eluate at -80°C for further bio-molecular analyses.

RNA of each sample was quantified on Infinite M200 Pro instrument with NanoQuant plate (Tecan, Trading AG, Switzerland).

**Viral Genome Amplification**

Reagents

- LunaScript® RT SuperMix Kit (New England Biolabs, Ipswich, MA), consisted of:
- LunaScript® RT SuperMix
- No-RT Control Mix
- Nuclease-free Water
- Q5® High-Fidelity 2X Master Mix (New England Biolabs, Ipswich, MA) (includes Q5 High-Fidelity DNA Polymerase, dNTPs, and Mg++in a broad-use buffer)
- PoolOne and PoolTwo primers pool (oPools™ Oligo Pools, IDT, Integrated DNA Technologies, Inc., Coralville,USA), used at a concentration of 10 uM.

Equipment:

- Sterile, nuclease-free microcentrifuge tubes
- Sterile, RNase-free pipette tips
- Vortex mixer
- Microcentrifuge
- Thermal-cycler

Protocol

**Retrotrascription**

1. Gently mix the LunaScript® RT SuperMix.
2. In a sterile, nuclease free microcentrifuge tube, add the following components: 4 uL of LunaScript® RT SuperMix and 6 uL of RNase-free water, to reach a final volume of 10 uL.
3. Add 10 uL of extracted RNA to the reaction mix.
4. Mix by vortexing and centrifuge briefly.
5. Place the reaction tube into a thermal-cycler programmed as follow: 25°C for 2 min, 55°C for 20 min, 95° for 1 min.

**Amplification**

1. Gently mix the Q5® High-Fidelity 2X Master Mix and PoolOne and PoolTwo primer tubes.
2. In two different microcentrifuge tubes, add 12.5 uL of Q5® High-Fidelity 2X Master Mix, 2.5 uL of PoolOne (10 uM) or 2.5 uL of PoolTwo (10 uM) and 4uL of RNase free water to reach a final volume of 19 uL.
3. Add 6 uL of retro-transcribed to both reaction mix.
4. Mix by vortexing and centrifuge briefly.
5. Place the reaction tubes into the same thermo-cycler with the following temperatures: 98°C for 30 sec, followed by 35 cycles of 95° C for 15 s and 65° for 5 min.

The amplicons were checked on the 4200 TapeStation System (Agilent, Santa Clara, US), to detect the amplified peak at 400 bp.

**Amplicon purification**

Reagents

- 1.8x Agencourt AMPure XP Beads (Beckman Coulter), in proportion 1:1.8 to the sample
- Resuspension buffer ()
- Ethanol 80%

Equipment:

- Magnetic stand ()
- 96-well plate ()
- Microcentrifuge
- Vortex mixer
- Sterile, RNase-free pipette tips

Protocol

1. Briefly centrifuge the entire contents of PoolOne and PoolTwo PCR reactions for each biological sample and combine them into a single tube, to reach a final volume of 50 uL. Mix by vortex mixer and centrifuge briefly again.
2. Add 50 uL 1.8x Agencourt AMPure XP Beads (previously vortex the solution to ensure the beads are well resuspended) to each of the 50 uL combined sample. Pipet up and down thoroughly to mix.
3. Incubate for 5 min at room temperature.
4. Place the plate onto a magnetic rack.
5. After the solution has cleared (2 min or longer), carefully remove the supernatant without discarding the beads.
6. With the plate still on the magnetic rack, add 200 uL of 80% ethanol.
7. Carefully remove and discard the wash. Repeat for a total of 2 ethanol washes.
8. Completely remove any residual ethanol with a 10 uL pipette after second wash. Air dry at room temperature for 1 min or until the pellet loses its shine.
9. Remove the tube from the magnetic rack and elute the DNA from the beads by adding 30 uL Resuspension Buffer. Mix well by pipetting and incubate for 2 min.
10. Return the plate on the magnetic rack until the solution has cleared.
11. Transfer the solution of all samples into a clean plate.

**Library preparation of Whole Genome using Next Generation Sequencing**

Reagents

Illumina DNA Prep and IDT ILMN DNA/RNA Index kit (Illumina, San Diego, CA, USA) for 96 samples, consisted of:

Equipment

- 96-plate magnetic stand
- 96-plate benchtop centrifuge
- Benchtop microcentrifuge
- Thermal cycler
- Dry block heater
- Vortex mixer
- Shaker
- Microseal

Protocol

DNA Tagmentation

This process fragments and tags the DNA with adapter sequences.

1. Transfer between 2 uL and 30 uL of DNA into the wells of a 96-well PCR plate, so that the total input amount (ng) is within the desired range.
2. Add nuclease-free water to the DNA samples to reach the total volume to 30 uL
3. Bring to room temperature and vortex to mix the BLT and TB1 consumables.
4. Prepare tagmentation master mix using 11 uL of BLT and 11 uL of TB1 for each reaction.
5. Vortex the tagmentation master mix thoroughly to make sure the beads are evenly resuspended in buffer.
6. Transfer 20 uL of tagmentation master mix to each well containing a sample.
7. Resuspend reaction mix by pipetting up and down.
8. Seal with Microseal “B” and centrifuge the plate. Place on the preprogrammed thermal cycler consisted of 55°C for 15 min and 10°C hold, preheating the lid at 100°C.

Post Tagmentation Cleanup

This step washes the adapter-tagged DNA on the BLT before PCR amplification.

1. Bring to room temperature the TWB and TSB consumable. If the TSB has any precipitates, heat the buffer at 37° for 10 min, and vortex until they dissolve.
2. Add 10 uL of TSB to the tagmentation reaction.
3. Gently pipette up and down the entire volume to well resuspend the beads.
4. Seal the plate and incubate at 37°C for 15 min on a thermal cycler with heated lid set at 100°C and volume reaction of 60 uL. Then hold at 10°C.
5. Place the plate on the magnetic stand for 3 min or until solution is clear.
6. Remove and discard the supernatant.
7. Remove the plate from the magnetic stand and add 100 uL of TWB. Gently pipette up and down the entire volume to well resuspend the beads.
8. Place the plate on the magnetic stand for 3 min of until solution is clear.
9. Remove and discard the supernatant.
10. Repeat steps 7 through 10 for a total of 2 washes.
11. Remove the plate from the magnetic stand and add 100 uL of TWB. Gently pipette up and down the entire volume to well resuspend the beads.
12. Seal the plate, place on the magnetic stand and incubate for at least 3 min or until clear.

Amplify Tagmented DNA

This step amplifies the tagmented DNA using a limited-cycle PCR program.

1. Thaw on ice the EPM buffer. Invert to mix and briefly centrifuge.
2. Thaw at room temperature the DNA Adapters. For index tubes, vortex to mix and briefly centrifuge. For plate, spin briefly before use.
3. Prepare the PCR master mix combining 22 uL of EPM and 22 uL of nuclease-free water for each sample.
4. Vortex and spin briefly the PCR master mix.
5. Remove the third TWB wash from samples while on the magnetic stand, discarding any excess liquid from the plate.
6. Remove the plate from the magnetic stand.
7. Add 40 uL of the PCR master mix to each sample. Gently pipette mix to well resuspend the beads.
8. Add 5 uL i5 adapter and 5 uL i7 adapter to each sample.
9. Pipette mix several times to mix the entire reaction volume.
10. Seal the plate, place in the thermal cycler programmed as follow: 68°C for 3 min, 98°C for 3 min, 12 cycles of 98°C for 45 sec, 62°C for 30 sec and 68°C for 2 min. 68 °C for 2 min, 10°C hold. The number of PCR cycles was chosen depending on expected initial DNA input (1-9 ng).
11. Remove plate from the thermal cycler and centrifuge at 280 g for 1 min.

Clean up Library

1. Let stand at room temperature the PB for 30 min. Vortex and invert to mix.
2. Thaw and bring to room temperature the RSB. Vortex to mix.
3. Place the plate on the magnetic stand for 5 min or until the supernatant is clear.
4. Transfer 45 uL of the PC supernatant into a fresh Midi plate.
5. Prepare the master mix of diluited SPB mixing 45 uL of PB and 40 uL of nuclease-free water for each sample.
6. Vortex the diluited PB master mix thoroughly and add 85 uL mix to each PCR product.
7. Pipette mix a minimum of 10 times or until thoroughly mixed.
8. Seal the plate and incubate at room temperature for 5 min.
9. Place the Midi plate on a magnetic stand for 5 min or until supernatant is clear.
10. During incubation, vortex the PB (undiluted stock tube) thoroughly, and then add 15 uL to each well in a new plate.
11. Transfer 125 uL of supernatant from the first Midi plate into a second plate (containing 15 uL of PB) and pipette mix 10 times.
12. Seal the plate and incubate at room temperature for 5 min.
13. Place the plate on a magnetic stand for 5 min or until clear.
14. Remove and discard supernatant without disrupting the beads.
15. With the plate on the magnet, add 200 uL of fresh 80% ethanol without mixing and incubate for 30 sec.
16. Remove the ethanol.
17. Repeat steps 15 and 16 for a total of 2 washes. Remove any excess liquid from the plate.
18. Air-dry on the magnetic stand for about 5 min until dry.
19. Remove the plate from che magnetic stand and add 32 uL of RSB to the beads. Pipette mix until resuspended.
20. Incubate at room temperature for 2 min.
21. Place the plate back on the magnetic stand for 2 min or until clear.
22. Transfer 30 uL of the supernatant into a new 96-well PCR plate.

Pooling Library

Library concentration was determined with the Invitrogen Qubit 2.0 Fluorometer and the Quant-iT Picogreen dsDNA kit (Fisher Thermo Scientific, Waltham, MA).

The molarity of the pooled library was calculated using following formula provided by Illumina.

The library was denaturized and diluted according to the input library.

Detailed Procedure

WNV RNA was manually extracted from bird homogenates and mosquito pools by using QIAMP viral RNA mini Kit (Qiagen GmbH, Germany), according to the manufacturer's instructions, described in detail in paragraph XX. After the extraction, RNA was quantified on Infinite M200 Pro instrument with NanoQuant plate (Tecan, Trading AG, Switzerland) and only the RNA of positive WNV-2 samples has been retro-transcribed to cDNA using the LunaScript® RT SuperMix Kit (New England Biolabs, Ipswich, MA) and amplified by an home-made protocol using primer pools generating 400 bp amplicons. Primer for each pool were generated on PrimalScheme online tool, following the instructions provided by the web site (https://primalscheme.com/). The primer sequences produced by the program for the two pools were screened on dataset of complete WNV-2 genomes (two isolates from 2004 to 2018) downloaded from GeneBank to identify the presence of nucleotide sites that need to be degenerated. Each pool was generated using the oPools™ Oligo Pools tool (IDT, Integrated DNA Technologies, Inc., Coralville,USA) and was diluited to a concentration of 10 uM, following the instruction of the manufacturer. The PCR reaction was performed in a 25 μL of mix for each pool containing 6 μL of template cDNA, 2.5 μL of each 10 μM primer pool, 12.5 of Q5® Hot Start High-Fidelity 2X Master Mix (New England Biolabs, Ipswich, MA) and 4 μL of Nuclease-free water. Thermocycling conditions consisted of 30 s at 98° C, followed by 35 cycles of 95° C for 15 s and 65° for 5 min. The result of the PCR products was checked on the 4200 TapeStation System (Agilent, Santa Clara, US), to detect the exact amplified peak at 400 bp and then purified using a 1.8x volume of Agencourt AMPure XP Beads (Beckman Coulter). Amplicons were pooled at equal concentration and libraries were prepared using Illumina DNA Prep and IDT ILMN DNA/RNA Index kit (Illumina, San Diego, CA). Library concentration was determined with the Invitrogen Quant-iT Picogreen dsDNA assay (Fisher Thermo Scientific, Waltham, MA). Resulting libraries were normalized and pooled for sequencing using a 2×200 cycle paired-end sequencing protocol. FASTQ files were generated from MiSeq Reporter (Illumina) and the paired reads were imported to Geneious Prime software v. 11.1 (<https://www.geneious.com/>). The reads generated, were mapped to a WNV-2 reference sequence (NC_001563.2) to generate consensus sequences, by using the “Map to reference” command in the “Assembly and Mapping” section.
